# SiFit: inferring tumor trees from single-cell sequencing data under finite-sites models

**DOI:** 10.1186/s13059-017-1311-2

**Published:** 2017-09-19

**Authors:** Hamim Zafar, Anthony Tzen, Nicholas Navin, Ken Chen, Luay Nakhleh

**Affiliations:** 1 Department of Computer Science, Rice University, Houston, Texas USA; 2 Department of Bioinformatics and Computational Biology, University of Texas M.D. Anderson Cancer Center, Houston, Texas USA; 3 Department of Genetics, University of Texas M.D. Anderson Cancer Center, Houston, Texas USA

**Keywords:** Tumor evolution, Intra-tumor heterogeneity, Single-cell sequencing, Finite-sites model, Phylogenetic tree

## Abstract

Single-cell sequencing enables the inference of tumor phylogenies that provide insights on intra-tumor heterogeneity and evolutionary trajectories. Recently introduced methods perform this task under the infinite-sites assumption, violations of which, due to chromosomal deletions and loss of heterozygosity, necessitate the development of inference methods that utilize finite-sites models. We propose a statistical inference method for tumor phylogenies from noisy single-cell sequencing data under a finite-sites model. The performance of our method on synthetic and experimental data sets from two colorectal cancer patients to trace evolutionary lineages in primary and metastatic tumors suggests that employing a finite-sites model leads to improved inference of tumor phylogenies.

**Electronic supplementary material:**

The online version of this article (doi:10.1186/s13059-017-1311-2) contains supplementary material, which is available to authorized users.

## Background

Intra-tumor heterogeneity, which is caused by a combination of mutation and selection [1–4], poses significant challenges to the diagnosis and clinical therapy of cancer [5–8]. This heterogeneity can be readily elucidated and understood if the evolutionary history of the tumor cells is known. This knowledge, alas, is not available, since genomic data is most often collected from one snapshot during the evolution of the tumor’s constituent cells. Consequently, using computational methods that reconstruct the tumor phylogeny from sequence data is the approach of choice. However, while intra-tumor heterogeneity has been widely studied, the inference of a tumor’s evolutionary history remains a daunting task.

Most studies to date have relied on bulk high-throughput sequencing data, which represents DNA extracted from a tissue consisting of millions of cells [9–13]. As a result, the admixture signal obtained from such data represents an average of all the distinct subpopulations present in the tumor [14]. This ambiguity makes it difficult to identify the lineage of the tumor from the mixture. In such cases, phylogenetic reconstruction requires a deconvolution of the admixture signal to identify the taxa of the tree [15–17]. This type of data is low resolution and cannot depict the cell-to-cell variability that is needed for inference of tumor evolution [14, 18]. Another approach for resolving intra-tumor heterogeneity and reconstructing tumor phylogeny is multi-region sequencing, in which DNA sampled from multiple spatially separated regions of the tumor are sequenced [19, 20]. However, this approach is restricted to when the subpopulations are geographically segregated and it cannot resolve spatially intermixed heterogeneity [21].

### Single-cell DNA sequencing: promises and challenges

With the advent of single-cell DNA sequencing (SCS) technologies, high-resolution data are becoming available, which promises to resolve intra-tumor heterogeneity to a single-cell level [14, 18, 22–25]. These technologies provide sequencing data from single cells, thus allowing for the reconstruction of the cell lineage tree. However, the high error rates associated with SCS data significantly complicate this task.

The whole-genome amplification (WGA) process, a crucial step in producing SCS data, introduces different types of noise that result in erroneous genotype inferences. The prominent WGA errors include: allelic dropout (ADO) errors, false positive errors (FPs), non-uniform-coverage distribution, and low-coverage regions [14]. ADO is a prominent error in SCS data and it contributes a considerable number of false negatives (FNs) in point mutation data sets. ADO is responsible for falsely representing the heterozygous genotypes as homozygous ones and the extent of such errors varies from 0.0972 to 0.43 as reported in different SCS-based studies [22–26]. Even though variant callers have been proposed for reducing ADO errors [27], the extent of such errors is still large. Different SCS studies have reported FP rates varying from 1.2×10^−6^ to 6.7×10^−5^ [22–26], the number of occurrences of which can essentially exceed the number of true somatic mutations. Often a consensus-based approach is taken to reduce the number of FP errors [26–28], in which, only variants observed in more than one single cell are considered. The variants observed in only one single cell are treated as errors and removed. In doing so, this approach also removes the true biological variants unique to a cell, whereas sites of recurrent errors persist. Both ADO and coverage non-uniformity result in unobserved sites. Often more than 50% of the genotypes are reported as missing due to the low quality of SCS data and, thus, no information regarding the mutation status of that site is conveyed [22].

Another source of error in SCS data is cell doublets in which two or more cells are accidentally isolated instead of single cells. Cell-doublet error rates vary considerably depending on the isolation technology. Methods such as fluorescence-activated cell sorting have reported less than 1% cell-doublet error rates [29–31], while doublet rates for methods such as mouth pipetting and microdroplet encapsulation technologies range from 1 to 10% [22, 23, 32].

### Existing work

Single-cell-based studies for delineating tumor phylogeny rely on single-cell somatic single-nucleotide variation (SNV) profiles, which are confounded by the technical errors in SCS. Even though such errors prohibit the use of classic phylogenetic approaches, many studies have used them. Distance-based methods like UPGMA and neighbor joining have been used by Yu et al. [33] and Xu et al.[23], respectively. Eirew et al. [34] used a popular Bayesian phylogenetic inference tool, MrBayes [35], for inferring evolutionary history. However, none of these methods account for the SCS-specific errors.

BitPhylogeny [36] is a non-parametric Bayesian approach that uses a tree-structured mixture model to infer intra-tumor phylogeny. Even though such an approach is valuable for identifying subclones from bulk sequencing data, it is not suitable in the context of present-day single-cell data sets [24, 26, 33, 37], which do not provide sufficient data for the mixture model to converge to the target distribution [38]. Furthermore, BitPhylogeny is a flexible framework that can fit different data types but does not specifically model single-cell errors.

SCITE [39] and OncoNEM [38] are two computational tools that were specifically designed for inferring tumor evolution from SCS data. SCITE is a Markov chain Monte Carlo (MCMC) algorithm that allows one to infer a maximum likelihood (ML) tree from the imperfect genotype matrix of SCS. It infers the evolutionary history as a mutation tree, as proposed by Kim and Simon [40]. A mutation tree shows the chronological order of the mutations that occur during tumor development. OncoNEM is a likelihood-based method that employs a heuristic search algorithm to find the ML clonal tree, a condensed tree that represents the evolutionary relationship between the subpopulations in the data. OncoNEM clusters the cells together into clones and also infers unobserved populations that can improve the likelihood. Both methods probabilistically account for technical errors in SCS data and can also estimate the error rates of SCS data. However, both SCITE and OncoNEM suffer by making inferences under the infinite-sites assumption, which posits that each site in the data set mutates at most once during the evolutionary history [41] and the taxa form a perfect phylogeny [42]. This assumption is often violated in human tumors due to different events such as chromosomal deletions, loss of heterozygosity (LOH), and convergent evolution [43]. Furthermore, OncoNEM infers clonal trees where cell-to-cell evolution is not displayed, and SCITE is concerned with the order of mutation in the tree but not the lineage of single cells. To the best of our knowledge, there is no method that infers a phylogenetic tree from SCS data under a finite-sites model of evolution while accounting for the technical errors in SCS.

### SiFit

Here we propose SiFit, a likelihood-based approach for inferring tumor trees from imperfect SCS genotype data with potentially missing entries, under a finite-sites model of evolution. To account for the errors in SCS, SiFit extends the error model of SCITE and OncoNEM. This extension accommodates for the possible genotypes that are excluded by the infinite-sites model. SiFit employs a finite-sites model of evolution that accounts for the effects of deletion, LOH, and point mutations on the genomic sites via transition probabilities between genotype states. SiFit employs a heuristic search algorithm to find the phylogenetic tree that is most likely to produce the observed SCS data. We evaluate SiFit on a comprehensive set of simulated data, where it performs superior to the existing methods in terms of tree reconstruction. The application of SiFit to experimental data sets shows how the infinite-sites assumption is violated in real SCS data and how SiFit’s reconstructed tumor phylogenies are more comprehensive compared to phylogenies reconstructed under the infinite-sites assumption. SiFit achieves a major advance in understanding tumor phylogenies from single cells and is applicable to a wide variety of available SCS data sets.

## Results and discussion

### Overview of SiFit

We start with a brief explanation of how SiFit infers a tumor phylogeny from noisy genotype data obtained from SCS. The input data consist of the following:

1. An *n*×*m* genotype matrix, which contains the observed genotypes for *m* single cells at *n* different loci. The genotype matrix can be binary or ternary depending on the data.
2. The FP rate (*α*) and FN rate (*β*). These error parameters can be learned from the data.

SiFit includes (1) a finite-sites model of tumor evolution and an error model for SCS, based on which the likelihood score of a candidate phylogenetic tree and error rate can be quantified and (2) a heuristic algorithm for exploring the joint space of trees and error rates in search of optimal parameters.

SiFit outputs a phylogenetic tree describing the evolutionary relationship between the single cells and the estimated error rates. The single cells are placed at the leaves of the phylogenetic tree. A more detailed technical description of SiFit can be found in “Methods” section.

#### Phylogenetic trees and model of tumor evolution

We assume that the observed single cells evolved according to an underlying phylogenetic tree. A phylogeny or phylogenetic tree represents the genealogical relationship among genes, species, populations, etc. [44]. In the context of a tumor, it is a rooted binary tree that represents the genealogical relationship among a set of cells. The sequenced single cells are placed at the leaves of the phylogenetic tree. We also assume that the cells evolve according to a finite-sites model along the branches of the tree.

The *n*×*m* true genotype matrix *G* contains the true genotypes of *m* single cells at *n* different loci. If the data contain information only about the presence or absence of a mutation at a locus, the matrix is binary, where the absence or presence of a mutation is represented by a 0 or 1 at the entry *G*(*i*,*j*), respectively. Assuming the cells to be diploid, if the data differentiates between heterozygous and homozygous mutations, the genotype matrix is ternary, where a 0, 1, or 2 at entry *G*(*i*,*j*) denotes a homozygous reference or a heterozygous or homozygous non-reference genotype, respectively. Heterozygous or homozygous non-reference genotypes represent mutations. This ternary representation facilitates the use of a mutation profile from modern variant-calling algorithms (e.g., Monovar [27] and GATK [45]), which report the mutation status of a sample in terms of genotypes.

To accommodate SCS data, we develop a finite-sites model of evolution ($\mathcal {M}$) that accounts for the effects of point mutations, deletions, and LOH on genomic sites. The finite-sites model of evolution encompasses a continuous-time Markov chain that assigns a transition probability for one genotype state changing to another along a branch of length *t*. The value of the transition probabilities depends on the branch length (*t*) and the parameters ($\mathcal {M}_{\lambda }$) of the model of evolution (see “Methods” section for details). By assigning a finite probability for all possible genotype transitions, this finite-sites model of evolution enables us to account for convergent evolution or reversal of genotypes that are excluded by methods that make the infinite-sites assumption (SCITE and OncoNEM). OncoNEM also assumes only binary data and does not differentiate between heterozygous and homozygous mutations. This binarization of data might result in loss of information for a data set with ternary genotypes, since heterozygous and homozygous non-reference genotypes cannot be distinguished when data is binarized. On the other hand, SCITE assumes that the observation of a homozygous non-reference genotype is due to technical errors only. These assumptions follow from using the infinite-sites model and are not made by SiFit.

SCITE also removes the mutations that are present in all cells or in one cell as non-informative in tree reconstruction. SiFit does not remove such mutations as these can be informative in the computation of the likelihood under a finite-sites model of evolution.

#### Model of single-cell errors

The observed genotype matrix, denoted by *D*, is an imperfect noisy version of the true genotype matrix *G*. The FP errors and the FN errors are responsible for adding noise in the observed genotype matrix. Considering binary genotype data, FP errors result in observing a 1 with probability *α* when the true genotype is 0. Similarly, due to FN errors, with probability *β*, we will observe a 0, instead of a 1. These relationships between the true and observed genotype matrices are given by

1 $$ \operatorname{Pr}(D_{i,j}|G_{i,j}) = \left\{ \begin{array}{lll} 1 - \alpha,\,\,\,\, & \text{if }\,\, D_{i,j} = 0, G_{i,j} = 0, \\ \beta,\,\,\,\, & \text{if }\,\, D_{i,j} = 0, G_{i,j} = 1, \\ \alpha,\,\,\,\, & \text{if }\,\, D_{i,j} = 1, G_{i,j} = 0, \\ 1 - \beta,\,\,\,\, & \text{if }\,\, D_{i,j} = 1, G_{i,j} = 1. \end{array}\right.  $$

The error model for ternary data is described in detail in “Methods” section. The observed genotype matrix can also have missing data because of the uneven coverage of SCS. SiFit handles missing data by marginalizing over possible genotypes (see “Methods” section for details).

#### Tree likelihood

A phylogenetic tree, $\mathcal {T} = (T, \mathbf {t})$, consists of a tree topology *T* and a vector of the branch lengths, **t**. Assuming the technical errors to be independent of each other and that sites evolve independently, the likelihood of a phylogenetic tree ($\mathcal {T}$), the error rates (***θ***=(*α*,*β*)), and the parameters of the model of evolution ($\mathcal {M}_{\lambda }$) are given by

2 $$ {}\begin{aligned} \mathcal{L}(\mathcal{T}, \boldsymbol{\theta}, \mathcal{M}_{\lambda}) = \operatorname{Pr}(D|\mathcal{T}, \boldsymbol{\theta}, \mathcal{M}_{\lambda}) = \prod_{i=1}^{n} \operatorname{Pr}(D_{i}|\mathcal{T}, \boldsymbol{\theta}, \mathcal{M}_{\lambda}), \end{aligned}  $$

where *D*_*i*_ is the observed data at site *i*. It is a vector with *m* values corresponding to *m* single cells. The likelihood calculation for a particular site is described in detail in “Methods” section. The ML estimate is obtained from

3 $$ (\mathcal{T}, \boldsymbol{\theta}, \mathcal{M}_{\lambda})_{\text{ML}} = \underset{(\mathcal{T}, \boldsymbol{\theta}, \mathcal{M}_{\lambda})} {\text{argmax}}\hspace{1mm} \operatorname{Pr}(D|\mathcal{T}, \boldsymbol{\theta}, \mathcal{M}_{\lambda}).  $$

#### Heuristic search algorithm

Our model has three main components: the phylogenetic tree ($\mathcal {T}$), the error rates of single-cell data (***θ***), and the parameters of the model of evolution $(\mathcal {M}_{\lambda })$. The tree search space has (2*m*−3)!/2^*m*−2^(*m*−2)! discrete bifurcating tree topologies for *m* cells, and each topology has a continuous component for branch lengths. The overall search space also has a continuous component for error rates and model parameters along with the tree space. We designed a heuristic search algorithm to explore the joint search space to infer the ML configuration of phylogeny, error rates and evolution model parameters. In the joint $(\mathcal {T}, \boldsymbol {\theta }, \mathcal {M}_{\lambda })$ space, we consider three types of moves to propose a new configuration. In each type of move, one component is changed. Thus, from a current configuration $(\mathcal {T}, \boldsymbol {\theta }, \mathcal {M}_{\lambda })$, a new configuration of $(\mathcal {T'}, \boldsymbol {\theta }, \mathcal {M}_{\lambda })$, $(\mathcal {T}, \boldsymbol {\theta }', \mathcal {M}_{\lambda })$, or $(\mathcal {T}, \boldsymbol {\theta }, \mathcal {M}_{\lambda '})$ is proposed. The new configuration is heuristically accepted according to a ratio of likelihood. The search procedure terminates when the likelihood does not improve or the maximum number of iterations has been reached.

### Performance on simulated data

First, we evaluated the performance of SiFit on extensive simulated data sets. The simulation studies were aimed at analyzing SiFit’s accuracy in phylogeny inference under different experimental conditions. We also assessed SiFit’s ability to estimate the error rates and its robustness against increased error rates. We compared SiFit’s performance to three other methods. To analyze how the tree inference process degrades if the inference algorithm fails to account for the SCS errors, we chose a representative of the classic phylogeny inference methods as used by Eirew et al. [34]. Eirew et al. used MrBayes [35], a Bayesian phylogenetic inference method, which reports a set of trees drawn from the posterior distribution. Even though it was applied on SCS data, this method does not account for the errors in that data. The trees inferred from this method can be directly compared against the true trees. For MrBayes, we compute the average tree reconstruction error by averaging over all inferred trees. We also compared against SCITE [39] and OncoNEM [38], methods that infer tumor trees under the infinite-sites assumption. SCITE was designed to infer a mutation tree, but it can also infer a binary leaf-labeled tree, where the cells are the leaf labels and edges contain mutations. We used SCITE to infer the binary leaf-labeled tree from simulated data sets so that they can be directly compared against the true trees. Since, SCITE is an MCMC-based algorithm, occasionally it might report more than one optimal tree. In such cases, we measure the average accuracy over all the reported trees. OncoNEM infers a clonal tree, which cannot be directly compared against the simulated trees. OncoNEM first infers a cell lineage tree and then converts it to a clonal tree by clustering nodes. The cell lineage tree inferred by OncoNEM is a different representation of the clonal tree. We convert the cell lineage tree inferred from OncoNEM to an equivalent phylogenetic tree (potentially non-binary) by projecting the internal nodes to leaves (for details see “Methods” section), enabling us to compare OncoNEM results against true trees.

We use the tree reconstruction error for the performance metric. This measures the distance of the inferred tree from the true tree. The distance between two binary trees is measured in terms of the Robinson–Foulds (RF) distance [46], which counts the number of non-trivial bipartitions that are present in the inferred or the true tree but not in both trees. We normalize this count using the total number of bipartitions in the two trees. The output of SiFit, SCITE, and the Bayesian phylogenetic inference algorithm (MrBayes) is compared against the true tree in terms of the RF distance. The tree inferred by OncoNEM might be non-binary, so for OncoNEM trees, we separately computed the FP and FN distances between the true tree and the inferred tree. For binary trees with the same leaf set, the FP and FN distances are equal. For a non-binary tree, the FP and FN distances could differ from each other. “Methods” section gives the details of the tree reconstruction error metric for comparing trees.

#### Accuracy of phylogeny inference

To analyze the accuracy of SiFit’s tree inference, we simulated three sets of single-cell data with varying levels of doublet noise: (1) data sets without any doublet (*δ*=0), (2) data sets with 5% doublet rate (*δ*=0.05), and (3) data sets with 10% doublet rate (*δ*=0.1). For each setting, we simulated random binary phylogenetic trees for a varying number of leaves (single cells). The number of cells, i.e., leaves in the trees, *m*, was varied as *m*=50, *m*=100, and *m*=200. The number of sites, *n*, was varied as *n*=200, *n*=400, and *n*=600. For each combination of *δ*, *n*, and *m*, we generated ten data sets that were simulated from ten random trees. At the root of the tree, all sites have a homozygous reference genotype. The sequences are evolved along the branches of the tree starting from the root. In each branch of the tree, we simulate four types of events that can alter the genotype of a site: a new mutation, a deletion, LOH, and a recurrent point mutation (see “Methods” section for details). After evolving, the leaves have genotype sequences with true mutations. *m* genotype sequences corresponding to *m* single cells constitute the true genotype matrix. Errors are introduced into the true genotype matrix to simulate single-cell errors. For data sets with doublets, doublets are formed by merging the genotypes of two single cells (see “Methods” section) with probability *δ*. The FN rate for cell *c*, *β*_*c*_, is sampled from a normal distribution with mean *β*_mean_=0.2 and standard deviation *β*_sd_=*β*_mean_/10. FNs are introduced into the genotype matrix with probability *β*_*c*_ for cell *c*. We introduced FPs into the genotype matrix with error rate *α*=0.01 by converting homozygous reference genotypes to heterozygous genotypes with probability *α*. It is important to note that here the FP rate, *α*, is by definition different from the false discovery rate (FDR) reported in single-cell-based studies such as [22, 24, 26]. *α* here indicates the fraction of non-mutant sites that are reported as mutant in the observed genotype matrix, whereas the FDR reported in the aforementioned studies refers to the number of FP errors per sequenced base pair. For exome-sequencing studies, even a very small FDR (∼10^−5^) can lead to a large number of FP variants in the observed genotype matrix, making *α* much higher than the reported FDR. After adding noise, the imperfect genotype matrices were used as input to SiFit for learning the ML tree.

**Fig. 1:**
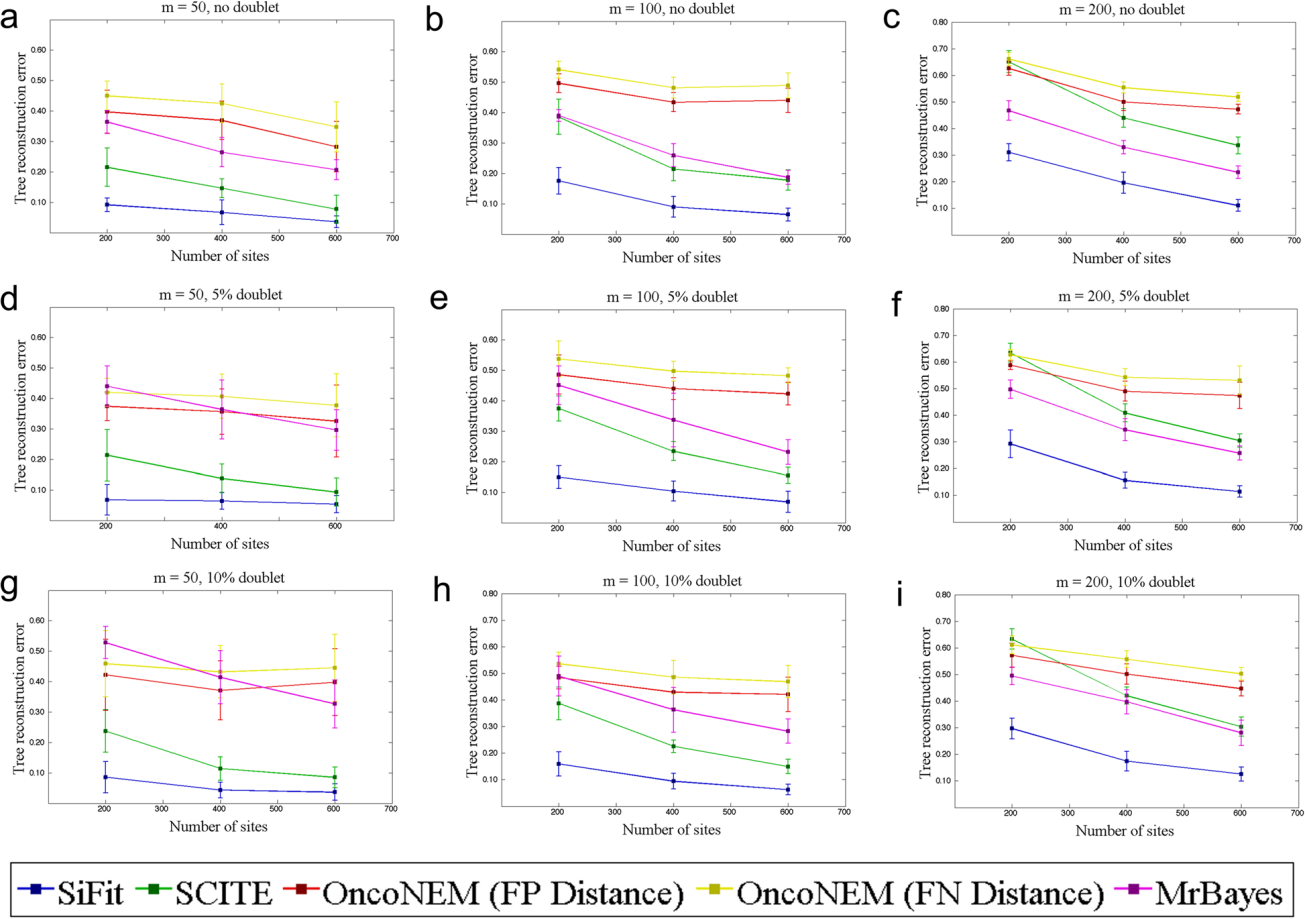
Performance comparison for data sets with a varying number of cells. SiFit’s tree reconstruction accuracy is compared against that of SCITE, OncoNEM, and MrBayes. The *y*-axis denotes the tree reconstruction error, which measures the distance of the inferred tree from the ground truth. Three points for *n*=200, *n*=400, and *n*=600 are plotted on the *x*-axis. In each case, the mean tree reconstruction error over ten data sets is plotted. The vertical error bar indicates the standard deviation of the tree reconstruction error over ten data sets. **a** Performance comparison for data sets with 50 cells and *δ*=0. **b** Performance comparison for data sets with 100 cells and *δ*=0. **c** Performance comparison for data sets with 200 cells and *δ*=0. **d** Performance comparison for data sets with 50 cells and *δ*=0.05. **e** Performance comparison for data sets with 100 cells and *δ*=0.05. **f** Performance comparison for data sets with 200 cells and *δ*=0.05. **g** Performance comparison for data sets with 50 cells and *δ*=0.1. **h** Performance comparison for data sets with 100 cells and *δ*=0.1. **i** Performance comparison for data sets with 200 cells and *δ*=0.1. For **d**–**i**, the tree reconstruction error is measured without considering the doublets in both true and inferred trees. FN false negative, FP false positive

SiFit’s tree inference accuracy was compared against three other methods. The same imperfect genotype matrix was used as input to SiFit and SCITE. For OncoNEM and MrBayes, the genotype matrices were binarized by converting the heterozygous and homozygous non-reference genotypes to 1, i.e., the presence of a mutation. The comparison is shown in Fig. 1, which shows the tree reconstruction error. For each value of *n*, the mean error metric over ten data sets is plotted along with the standard deviation as the error bar. For data sets without doublets, SiFit substantially outperforms the other three methods for all values of *m* and *n*. The performance of each algorithm except for OncoNEM improves as the value of *n* increases. The behavior of OncoNEM is different. For *m*=100, its accuracy decreases for *n*=600 compared to *n*=400. This might be because OncoNEM was developed for clonal tree inference and the effect of an additional number of sites cannot be observed in the equivalent phylogenetic tree unless they (the additional sites) are different across the clones. For data sets with a higher number of sites (*n*=600), SiFit was able to find either the true tree topology or a near-perfect tree topology for most of the data sets, demonstrating its ability to infer the correct trees given enough data.

For the data sets with doublets, we measured the tree reconstruction error in two ways: (1) doublets are removed from both the true tree and inferred tree and then the RF distance is calculated and (2) the RF distance is calculated between the true tree and inferred tree without any distinction of doublets. Since, doublets are a hybrid of two cells that belong to two places in the tree, measuring the tree reconstruction error as in (1) ensures that position of all the other cells except the doublets are properly inferred, whereas (2) measures the overall tree reconstruction error. Figure 1 compares the algorithms in terms of tree reconstruction error as described in (1). SiFit outperforms the other three methods for all values of *δ*, *m*, and *n*. The performance of SCITE and MrBayes is substantially affected by the presence of doublets, specifically for the data sets with a smaller number of mutations. In comparison, SiFit’s performance is much more robust in the presence of doublets while recovering the positions of the non-doublets in the tree. Even in terms of the overall tree reconstruction error (measured as described in (2)), SiFit performs better than the other algorithms for all simulation settings corresponding to different values of *δ*, *n*, and *m* (Additional file 1: Figure S1).

#### Inference with missing data

**Fig. 2:**
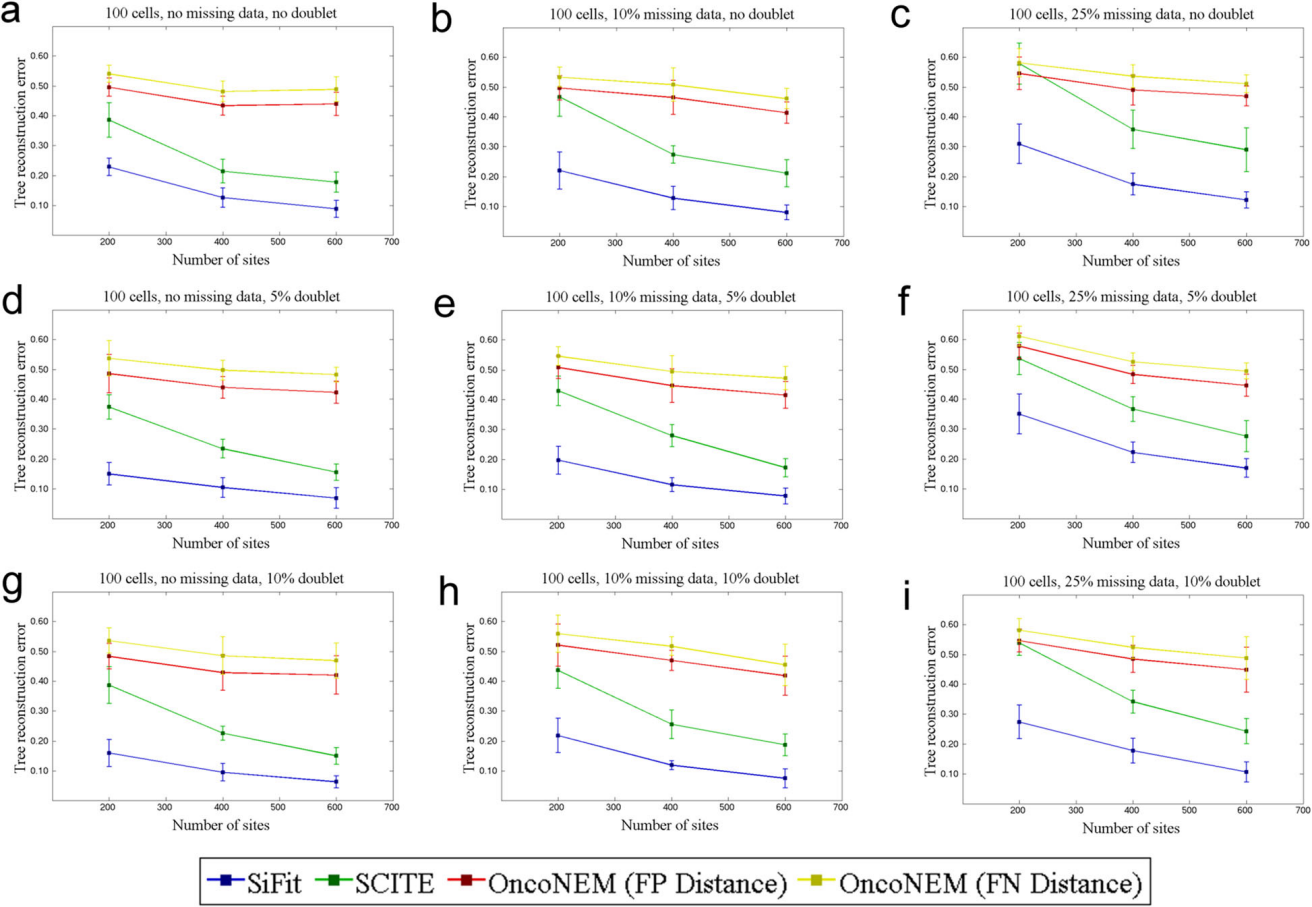
Performance comparison on data sets with missing data. SiFit’s tree reconstruction accuracy is compared against that of SCITE and OncoNEM on data sets with missing data. The *y*-axis denotes the tree reconstruction error, which measures the distance of the inferred tree from the ground truth. Three points for *n*=200, *n*=400, and *n*=600 are plotted on the *x*-axis. In each case, the mean tree reconstruction error over ten data sets is plotted. The vertical error bar indicates the standard deviation of the tree reconstruction error over ten data sets. **a** Comparison for data sets without any missing data and *δ*=0. **b** Comparison for data sets with 10% missing data and *δ*=0. **c** Comparison for data sets with 25% missing data and *δ*=0. **d** Comparison for data sets with without any missing data and *δ*=0.05. **e** Comparison for data sets with 10% missing data and *δ*=0.05. **f** Comparison for data sets with 25% missing data and *δ*=0.05. **g** Comparison for data sets without any missing data and *δ*=0.1. **h** Comparison for data sets with 10% missing data and *δ*=0.1. **i** Comparison for data sets with 25% missing data and *δ*=0.1. For **d**–**i**, the tree reconstruction error is measured without considering the doublets in both the true and inferred trees. FN false negative, FP false positive

Due to uneven coverage and amplification bias, current SCS data sets are challenged by missing data points where genotype states are unobserved. To investigate how missing data affect phylogeny reconstruction, we performed additional simulation experiments. For *m*=100 and *n*={200,400,600}, we generated data sets using the same error rates as before. For each combination of *δ*, *n*, and *m*, we generated ten data sets, for each of which, two other data sets with missing data of {10*%*,25*%*} were generated. To generate the data sets with missing data, the genotype information of sites was removed with probability 0.1, and 0.25 for missing data of {10*%*,25*%*}, respectively. SiFit’s results were compared against SCITE and OncoNEM. The results are shown in Fig. 2. For each value of *δ*, as the missing data rate increases from 0 to 25%, for each of the competing methods, we observe a steady increase in the tree reconstruction error.

For data sets without doublets (*δ*=0), irrespective of the percentage of missing data, SiFit performs substantially better than SCITE and OncoNEM. SiFit’s likelihood calculation treats each missing data point as contributing a marginal probability of 1, effectively making it equivalent to reducing the number of sites *n*. For the data sets with doublets, we measured the tree reconstruction error in two ways as described in the previous section. SiFit outperforms both SCITE and OncoNEM irrespective of the way the tree reconstruction error was measured (Fig. 2 and Additional file 1: Figure S2).

#### Robustness to increasing error rates

ADO is the major source of error in SCS data resulting in FNs [14]. To test the robustness of SiFit to an increase in FN rate *β*, we simulated data sets with increased FN rates. The number of cells *m* was set to 100 and the number of sites *n* was set to 400. The mean FN rate *β*_mean_ was varied from 0.2 to 0.4 in steps of 0.1, i.e., *β*_mean_∈{0.2,0.3,0.4}. The FN rate of cell *c*, *β*_*c*_, was sampled from a normal distribution as described in the previous experiment. The FP rate was set to *α*=0.01. With these settings, for each value of *β*_mean_∈{0.2,0.3,0.4}, ten data sets were simulated for phylogeny reconstruction.

**Fig. 3:**
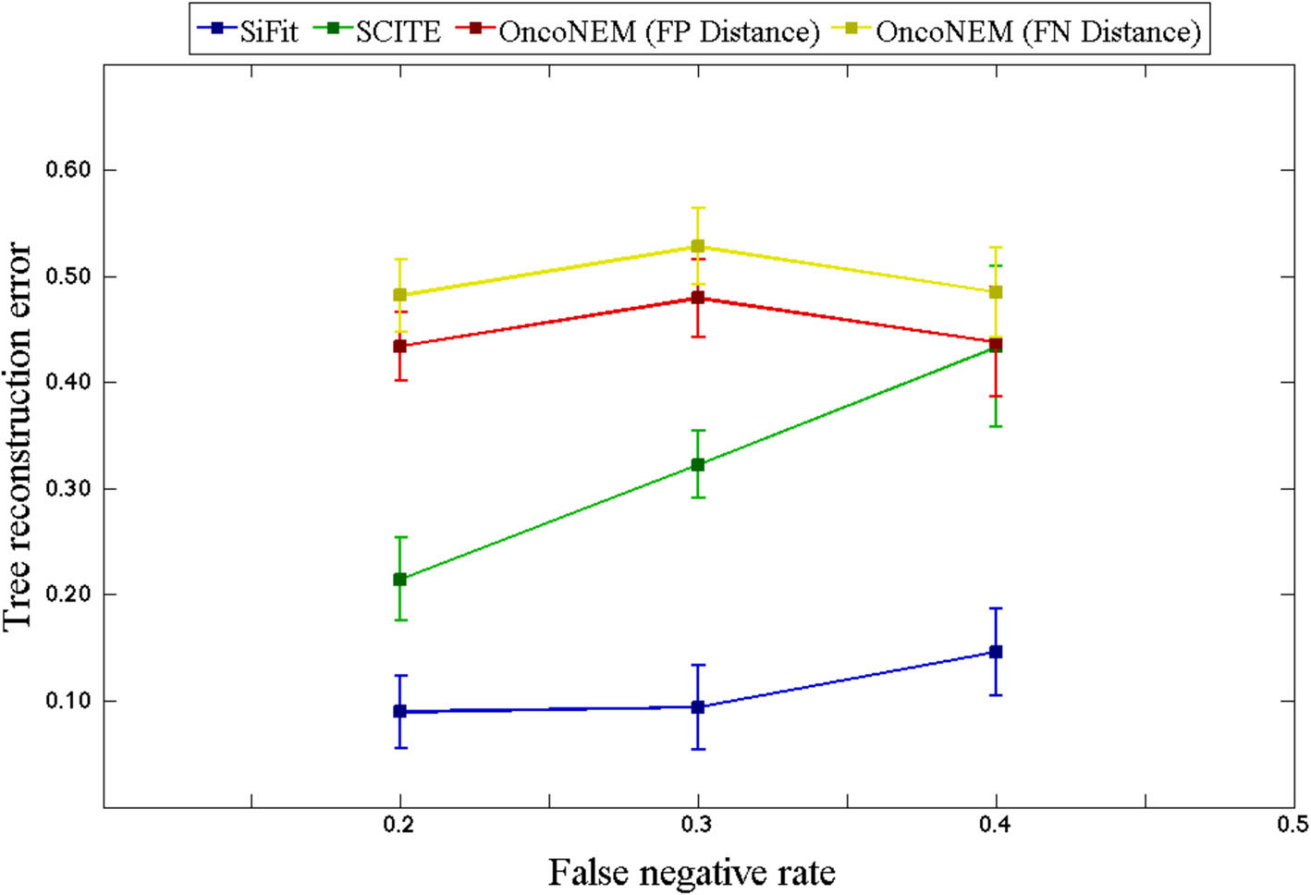
Effect of increase in error rates. SiFit’s tree reconstruction accuracy is compared against that of SCITE and OncoNEM for increasing FN rate. The *y*-axis denotes the tree reconstruction error, which measures the distance of the inferred tree from the ground truth. Four points corresponding to FN rate *β* = {0.2,0.3,0.4} are plotted. In each case, the mean tree reconstruction error over ten data sets is plotted. The vertical error bar indicates the standard deviation of the tree reconstruction error over ten data sets. FN false negative, FP false positive

The performance of SiFit was compared against SCITE and OncoNEM. For different settings of FN rates, SiFit consistently performs better than SCITE and OncoNEM by achieving the lowest tree reconstruction error (Fig. 3). For SCITE and SiFit, with the increase in the FN rate, the tree inference error increases. For OncoNEM, the tree reconstruction error first increases and then decreases. The rate of increase in tree reconstruction error for SiFit is also much lower compared to that of SCITE. This indicates SiFit’s higher robustness against amplification errors compared to SCITE. OncoNEM’s tree reconstruction error is higher than those of SCITE and SiFit for all values of the FN rate. For OncoNEM, binarization of the data leads to loss of information and it employs a grid search to learn the parameters before learning the optimal tree. This divisive sequential approach of learning may lead to a suboptimal solution if the initial solution gets stuck in local optima.

#### Estimation of error rates

In addition to the phylogenetic tree, SiFit also learns the error parameters from the data. To examine SiFit’s capability to estimate the FN rate from the data, we simulated 30 data sets from 30 random binary trees. For these data sets, the number of cells was set to 100, the number of sites was set to 400, and the FP rate was set to *α*=0.01. The FN rate *β* was varied from 0.1 to 0.4. These imperfect data matrices were given to SiFit for inference of the tree and FN rate.

**Fig. 4:**
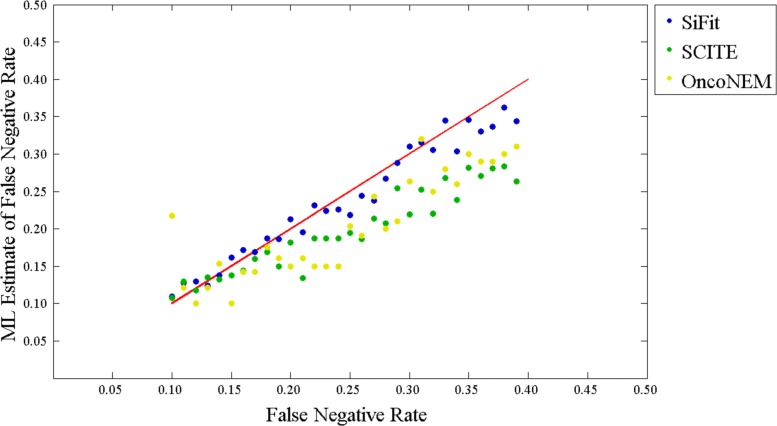
Estimation of error rates. The maximum likelihood estimate of the false negative rate is compared against the false negative rate used for generating the data. The red line represents the perfect estimate (the correlation coefficient is 1). The blue dots represent the estimates by SiFit, the green dots represent the estimates by SCITE, and the yellow dots correspond to estimates by OncoNEM. ML maximum likelihood

SiFit performed very well in estimating FN rate, as shown in Fig. 4. The ML values of *β* learned from the data were highly correlated (0.9843) to the ones that generated the data. This experiment demonstrates SiFit’s ability to infer error parameters from data.

SCITE and OncoNEM can also learn the FN rate from the data. To compare SiFit’s estimate of the error rate against those of OncoNEM and SCITE, we applied SCITE and OncoNEM to the same data sets for learning FN rates. SCITE’s performance (correlation 0.9622) was better than that of OncoNEM (correlation 0.8766) but SiFit was the best performer. Specifically, for data sets with a higher FN rate (>0.2), SiFit’s estimates were much better than those of SCITE and OncoNEM. This indicates a degree of robustness of SiFit in the presence of higher error rates compared to the other methods.

#### Run times

To measure the run time of SiFit, we simulated data sets containing different numbers of cells. The number of cells, i.e., leaves in the trees, *m*, was varied as *m*=100, *m*=200, and *m*=500. The number of sites *n* was varied as *n*=200 and *n*=400. The error rates were chosen as described in the previous experiments. For each combination of *m* and *n*, ten data sets were simulated. For each of these data sets, SiFit was run for 200,000 iterations in a node with 24 CPU cores (AMD 2.2 GHz). In each case, the average run time for 200,000 iterations was recorded (Additional file 1: Figure S3). For a fixed number of sites *n*, with the increase in the number of cells in the tree, SiFit’s run time increases almost linearly. This behavior is observed for both *n*=200 and *n*=400. This indicates that SiFit is scalable and will adopt well when future experiments generate sequencing data consisting of thousands of single cells. The theoretical computational complexity of SiFit is described in “Methods” section.

### Inference of tumor phylogeny from experimental SCS data

We applied SiFit to two experimental SCS data sets: exome sequencing from a non-hereditary colorectal cancer patient and high-throughput SCS from a metastatic colorectal cancer patient. From these data, we inferred the phylogenetic lineages of the tumor and ordered the chronology of mutations. These studies used different SCS methods and had different samples sizes and error rates. We selected them to show that SiFit is flexible and can be applied broadly to different single-cell mutation data sets.

#### Phylogenetic lineage of adenomatous polyps and colorectal cancer

SiFit was applied to single-cell exome sequencing data from a non-hereditary colorectal cancer [37] patient. The data set consisted of 61 single cells in total, with 35 cells sampled from colorectal cancer tissue, 13 from an adenomatous polyp tissue, and 13 from normal colorectal tissue. Variant calling resulted in the detection of 77 somatic SNVs from these 61 cells. In total, approximately 9.4*%* of the values were missing in the data set. The reported genotypes were binary values, representing the presence or absence of a mutation at the SNV sites (Additional file 1: Figure S4a).

To test whether the genotype matrix violates the infinite-sites assumption, we ran the four-gamete test. The four-gamete theorem states that an *m*×*n* binary matrix *M* has an undirected perfect phylogeny if and only if no pair of columns contain all four binary pairs (0,0; 0,1; 1,0; and 1,1), where *m* represents the number of taxa (leaves of the tree) and *n* represents genomic sites [47]. The perfect phylogeny model conveys the biological feature that every genomic site mutates at most once in the phylogeny [47] and that mutations are never lost. The existence of a perfect phylogeny shows that the data could fit the infinite-sites model of evolution. A violation of the four-gamete condition may indicate a potential deviation from the infinite-sites assumption. However, it is important to note that for SCS data, there could be more than one potential event leading to violation of the four-gamete test (see Additional file 1: Supplementary Note and Additional file 1: Figures S5 and S6 for more details). The binary mutation matrix from this colorectal patient violated the four-gamete test, with 1847 (out of 2926) pairs of SNV sites that contained all four binary pairs.

**Fig. 5:**
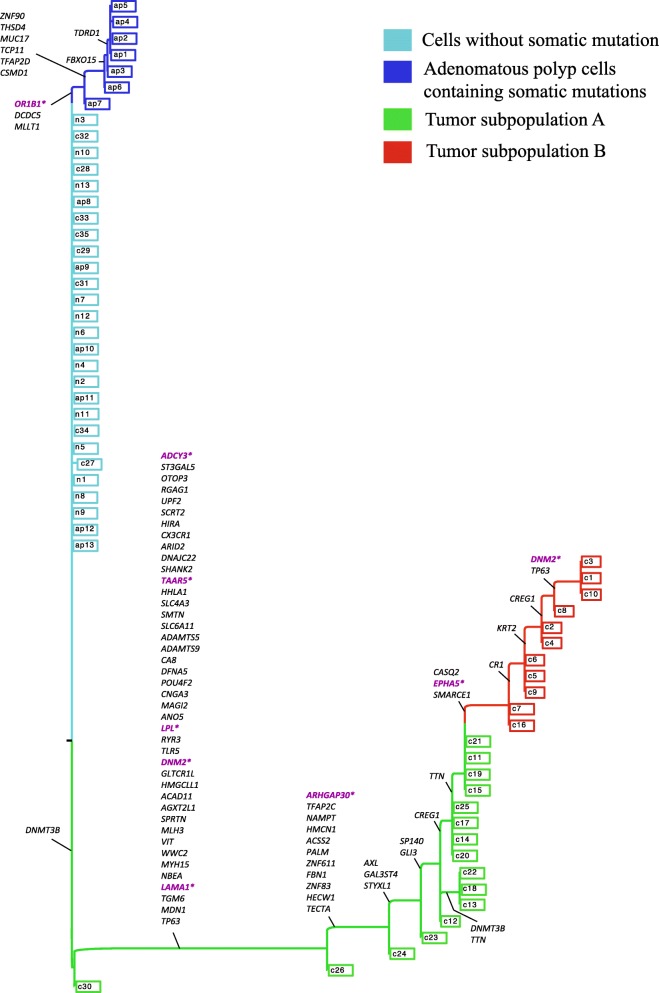
Maximum likelihood phylogenetic tree reconstructed by SiFit for adenomatous polyps and colorectal cancer. The leaves with legend marked with “n” are normal cells, leaves marked with “ap” are adenomatous polyp cells, and all other leaves are single tumor cells. Mutations are annotated on the branches of the tree. Important genes reported in the original study are marked in purple

The ML tree inferred by SiFit on 77 SNVs is shown in Fig. 5. The tree shows that the normal cells are placed very close to the root. In the original study, some of the adenomatous polyp cells were found to have no somatic mutations and were speculated to have derived from normal colorectal cells. In the tree inferred by SiFit, these cells (ap8–ap13) are accurately placed along with the normal cells. The original study also reported a set of cells from the cancer tissue as normal cells because they did not contain any somatic mutations. The tree inferred from SiFit placed these cells along with the normal cells, representing a completely independent lineage that likely initiated from a different originating cell.

We performed *k*-medoids clustering using the silhouette score (see “Methods” section for details) on the ML tree-based distance matrix. The cancer cells were clustered into two subpopulations (A and B). The chronological order of the mutations was inferred based on the inference of the mutation status of the internal nodes. We extended the algorithm in [48] for inferring ancestral sequences by accounting for single-cell-specific errors (see “Methods” section for details). This enabled us to find the ML solution for placing the mutations on the branches of the SiFit tree. Altogether, 53 clonal mutations occurred in the trunk of the tree, including mutations in *LAMA1* (PI3K-Akt signaling pathway) and *ADCY3* (FGFR signaling pathway). These clonal mutations are driver events that likely led to the expansion of subpopulation A. Subpopulation B emerged from subpopulation A by acquiring additional subclonal mutations in *EPHA5*, *CASQ2*, and *SMARCE1*. The SiFit tree also shows the evolution of the adenomatous polyp cells (marked in blue), which evolved from the normal cells by acquiring mutations in *OR1B1* (GPCR signaling pathway), *DCDC5*, and *MLLT1*. The adenomatous polyp cells evolved independently and further accumulated mutations in *CSMD1*, *FBXO15*, and *TCP11*. The tree inferred by SiFit represented the evolution of both the adenomatous polyp cells and the colorectal cancer cells and identified the order of the mutations that are associated with different signaling pathways and may have played a key role in the development of heterogeneity in this cancer patient.

To compare the results of SiFit with other algorithms, we also applied SCITE and OncoNEM on this data set. To enable a direct comparison, SCITE was used to infer a binary leaf-labeled tree, which is an ML solution with the single cells placed at the leaves of the tree. SCITE reported a single ML tree (*T*_SCITE_) from this data set (Additional file 1: Figure S7). We compared the tree inferred by SiFit (*T*_SiFit_) to the tree inferred by SCITE in terms of the likelihood value. Since the ML tree inferred by SCITE (*T*_SCITE_) does not have branch lengths, we cannot directly compute the likelihood value of *T*_SCITE_ using our likelihood function. Instead, we used the likelihood function of SCITE to compare the two trees. SCITE uses an expected mutation matrix defined by the mutation tree topology and sample attachments to compute the likelihood of a tree. After finding the ML placement of the mutations on the SiFit tree (*T*_SiFit_), we obtained the expected mutation matrix *E*, defined by *T*_SiFit_ and the annotated mutations on the branches of *T*_SiFit_ and then calculated its likelihood using Eq. 3 of [39]. This likelihood function of SCITE gives an edge to SCITE and is disadvantageous for SiFit because the branch lengths inferred by SiFit are ignored in this likelihood calculation. *T*_SiFit_ had a log-likelihood value of −632.5, which was substantially higher than the log-likelihood (−785.92) of *T*_SCITE_. This higher likelihood suggests that the tree inferred by SiFit explains the data better than that of SCITE on this experimental data set.

We used OncoNEM to infer the cell lineage tree (*T*_OncoNEM_) from this data set (Additional file 1: Figure S8). OncoNEM can also estimate the occurrence of mutations on the cell lineage tree based on posterior probability. Since, OncoNEM follows the infinite-sites assumption, if a cell in the lineage tree contains a mutation, all its descendants should have that mutation. Based on this principle and OncoNEM’s estimate of the occurrence of mutations, we can compute an expected mutation matrix that is defined by *T*_OncoNEM_. This enabled us to use the likelihood function of SCITE to compare *T*_OncoNEM_ against *T*_SiFit_. The log-likelihood value (−664.79) of *T*_OncoNEM_ was better than that of *T*_SCITE_ but it was worse than that of *T*_SiFit_. The higher likelihood of the tree inferred by SiFit compared to those of OncoNEM and SCITE suggests that the expected mutation matrix defined by SiFit’s tree inferred under a finite-sites model of evolution explains the data better than those of its contemporaries inferred under the infinite-sites assumption.

#### Phylogenetic lineage of a metastatic colorectal cancer patient

Next, we applied SiFit to infer the metastatic lineage of a colorectal cancer patient with a matched primary tumor and liver metastasis that was untreated. This data set consisted of highly-multiplexed SCS data [31] from 178 single cells using a 1000 cancer gene panel. Variant calling resulted in the detection of 16 somatic SNVs from these 178 cells [49]. The FP rate was estimated to be 1.52*%* and the FN rate was estimated to be 7.89*%*. In total, approximately 6.9*%* of the values were missing in the data set. The reported genotypes were binary values, representing the presence or absence of a mutation at the SNV sites (Additional file 1: Figure S4b).

Altogether, 104 (out of 120) pairs of SNV sites violated the four-gamete test, indicating the potential violation of the infinite-sites assumption.

**Fig. 6:**
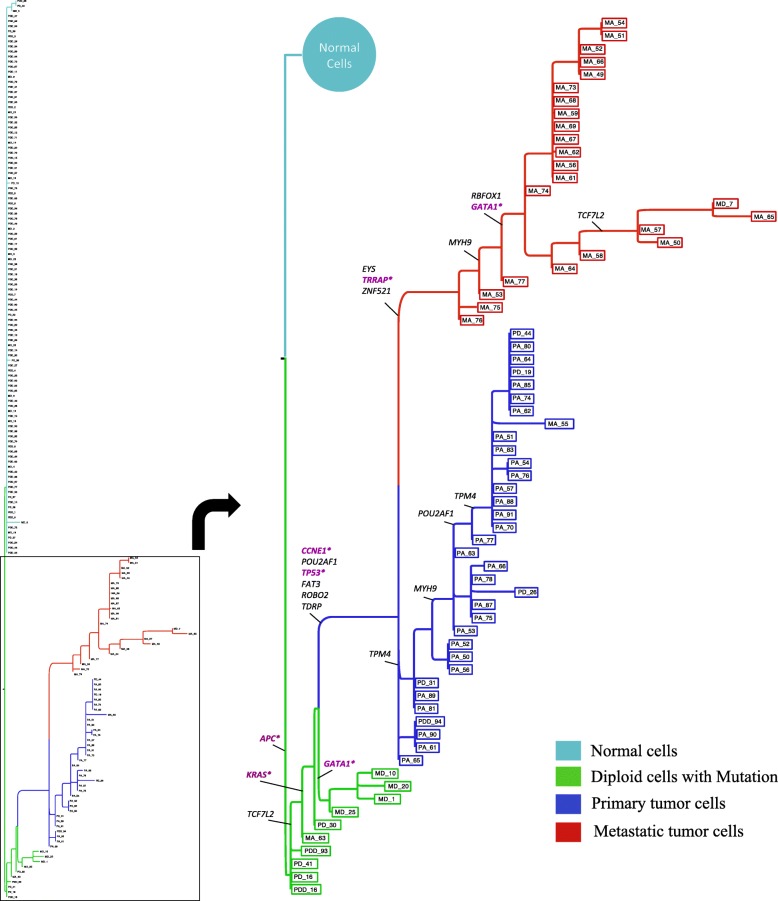
Maximum Likelihood phylogenetic tree reconstructed by SiFit for a metastatic colorectal cancer patient. Mutations are annotated on the branches of the tree. The cancer genes and tumor-suppressor genes are marked in purple

The ML tree inferred by SiFit from this data set is shown in Fig. 6. *k*-medoids clustering using the silhouette score on the ML tree-based distance matrix identified three subpopulations of somatically mutated cells along with the population of cells without mutations. The subpopulation of cells (marked in cyan) without mutations consisted mostly of diploid cells, suggesting they are normal stromal cells. The first somatic subpopulation (marked in green) consisted of mostly diploid cells. The second subpopulation (marked in blue) consisted of mostly primary aneuploid cells and a few diploid cells. The third subpopulation (marked in red) consisted of metastatic cells only. The chronological order of the mutations was inferred based on the ML placement of the mutations on the branches of the tree. Three diploid cells in the first subpopulation first acquired a heterozygous nonsense mutation in *APC*. This mutation was present in all the descendants (all primary and metastatic tumor cells), suggesting that this was the first mutation that initiated the tumor. Subsequently, mutations were acquired in the *KRAS* oncogene, the *TP53* tumor suppressor gene, and the *CCNE1* oncogene, which led to the expansion of the primary tumor mass. These primary tumor cells accumulated seven additional somatic mutations. In the later stages of the phylogeny, the accumulation of mutations in *EYS*, *ZNF521*, and *TRRAP* marked the point of metastatic divergence, after which tumor cells disseminated to the liver. Three more mutations occurred in *RBFOX1*, *GATA1*, and *MYH9*. The phylogeny also indicates potential losses of mutations, including *POU2AF1*, which was lost in 17 primary tumor cells, and the mutation in *TCF7L2*, which was lost in four metastatic tumor cells, but these losses did not mark any point of divergence, indicating they might be passenger mutations.

We also applied SCITE and OncoNEM on this data set. SCITE inferred a single binary leaf-labeled tree (*T*_SCITE_, shown in Additional file 1: Figure S9), which is the ML solution with a log-likelihood score of −387.68. To compute the likelihood of the tree (*T*_SiFit_) inferred by SiFit using SCITE’s likelihood function, we computed the expected mutation matrix *E* defined by *T*_SiFit_ using the ML placement of the mutations on its branches. *T*_SiFit_ had a higher value of the log-likelihood score (−201.63). OncoNEM was used to infer a cell lineage tree (*T*_OncoNEM_, shown in Additional file 1: Figure S10) from this data set. We also estimated the occurrence of mutations on *T*_OncoNEM_ based on the posterior probability values. This enabled us to calculate the likelihood of *T*_OncoNEM_ through the computation of the expected mutation defined by *T*_OncoNEM_. *T*_OncoNEM_ had a log-likelihood value of −349.95, which is worse than that of *T*_SiFit_. The higher likelihood value of *T*_SiFit_ on this data set suggests that the tree inferred by SiFit is superior to those of SCITE and OncoNEM in terms of explaining the data.

## Conclusions

Tumor phylogenies provide insight into the clonal substructure of tumors and the chronological order of mutations that arose during tumor progression. These lineages have direct applications in clinical oncology, for both diagnostic applications in measuring the amount of intra-tumor heterogeneity in tumors and for improving targeted therapy by helping oncologists identify mutations that are present in the majority of tumor cells. SCS data provides an unprecedented opportunity to reconstruct tumor phylogenies at the highest possible resolution. However, they are challenged by extensive technical errors that are introduced during genome amplification. In this paper, we introduced SiFit, a probabilistic method for recreating the evolutionary histories of tumors under a finite-sites model of evolution from imperfect mutation profiles of single cells. This likelihood-based approach can infer the ML phylogeny that best fits single-cell data sets with extensive technical noise. SiFit can also estimate the error rates of SCS experiments. SiFit employs a resilient error model that can account for various technical artifacts in SCS data, including ADO, FPs, and missing data. Our model is adaptable and can be easily extended to include position-specific error rates. SiFit also provides flexibility in choosing the model of evolution, for which we developed a finite-sites model of evolution that accounts for the effects of various events in tumor evolution such as point mutations, deletion, LOH, etc. in single-cell data sets. SiFit is robust to any variation in error rates and performs consistently with a varying number of cells in the data set, making it widely applicable to SCS data sets that vary in error rates and the number of cells sequenced.

The main difference between SiFit and existing methods, such as SCITE [39] and OncoNEM [38], is that SiFit introduces a finite-sites model of evolution. Both SCITE and OncoNEM make the infinite-sites assumption, which is frequently violated in convergent evolution or reversal of genotypes, events that occur in human tumors due to LOH and chromosomal deletions [43]. SiFit also makes use of high-resolution SCS data by utilizing the single cells as the taxonomic units of the reconstructed phylogenetic tree. On the other hand, SCITE reports a mutation tree in which the lineage of the cells is not shown. OncoNEM reports a clonal tree, which is a condensed tree with multiple cells clustered into a clone. This type of clonal clustering and the use of clones as the taxonomic units, though useful for finding genealogical relationships between clones, is low resolution as a clone represents a consensus of information from multiple single cells. The utilization of mutation information from each individual cell makes SiFit’s tree reconstruction method both robust and high resolution.

SiFit performs accurately, as evident from a comprehensive set of simulation studies that takes into account different aspects of modern SCS data sets by experimenting with a varying number of cells in the data set, a wide range of error rates, and different fractions of missing data. The simulation studies also demonstrated that SiFit substantially outperformed the state-of-the-art methods and is more robust to technical errors from WGA. We also applied SiFit to reconstruct the phylogeny for two experimental SCS tumor data sets from two patients with colorectal cancer, including one patient with a matched liver metastasis. SiFit accurately reconstructed the phylogenetic lineages of these tumors, and identified points in which subpopulations diverged from the main tumor lineages. These trees also provided insight into the order of mutations and the chronology in which they occurred during tumor progression.

SiFit’s phylogeny inference can potentially be improved by incorporating copy-number variations along with SNVs. Recent studies [50] indicate that copy number follows a punctuated evolutionary model and is likely to provide insight into possible LOH events and can facilitate tree inference. Such an approach has previously been used in the context of bulk sequencing data [16] and can be incorporated for SCS data under a finite-sites model of evolution. SiFit currently uses fixed error rates at every site. The error model can be further extended using position-specific error rates, where sites with lower-confidence mutations will have higher error rates and vice versa. The error model will have higher complexity in that situation and systematic model selection has to be performed. It is important to note that out of the three different types of events that could hint at a deviation from the infinite-sites assumption, SiFit currently models events (deletions, LOH, etc.) that affect the same genomic site more than once and the FP and FN errors in SCS data. The other potential source, cell doublets, are not explicitly included in SiFit’s error model. To include doublets in the error model, it will be necessary to move beyond the phylogenetic tree to phylogenetic networks, as doublets are an amalgamation of two separate genotypes and should be represented by a node of in-degree two. Another approach might be to treat them as a nuisance parameter and integrate them out during the likelihood calculation.

As SCS becomes more high-throughput [31, 51], enabling hundreds of cells to be analyzed in parallel at reduced cost and throughput, SiFit is poised to analyze the resulting large-scale data sets to understand the evolution of clones during tumor progression. SiFit represents a major step forward in understanding tumor phylogeny from SCS data and will have important translational applications for improving cancer diagnosis, treatment, and personalized therapy [14, 52]. Although the current study focused on cancer, SiFit can potentially also be applied to single-cell mutation profiles from a wide variety of fields, including immunology, neurobiology, microbiology, and tissue mosaicism [53]. These applications are expected to provide new insights into our understanding of cancer and other human diseases.

## Methods

### Input data

The input to SiFit is a matrix *D*_*n*×*m*_=(*D*_*ij*_) of observed genotypes, where *i*∈{1,...,*n*} denotes the index of genomic locus, *j*∈{1,...,*m*} is the index of the single cell, and *D*_*ij*_ is the observed genotype at the *i*th site of cell *j*. The genotype matrix can be binary or ternary depending on the representation of the data. For a binary matrix, *D*_*ij*_∈{0,1,*X*}, where 0, 1, and *X* denote the absence of a mutation, the presence of a mutation, and missing data, respectively. For a ternary matrix, *D*_*ij*_ can take values from the set {0,1,2,*X*}, where 0 denotes a homozygous reference genotype, 1 and 2 denote heterozygous and homozygous non-reference genotypes, respectively, and *X* denotes missing data.

### Model of single-cell errors

FP errors and FN errors are the two different types of noise that could be present in the genotype matrix. If *α* is the FP error rate and *β* is the FN error rate, then for a ternary genotype matrix, the relationship between the true and observed genotype matrices is given by

4 $$ \operatorname{Pr}(D_{i,j}|G_{i,j}) = \left\{\begin{array}{lll} 1 - \alpha - \frac{\alpha \beta}{2}, & \text{if }\,\, D_{i,j} = 0, G_{i,j} = 0, \\ \alpha, & \text{if }\,\, D_{i,j} = 1, G_{i,j} = 0, \\ \frac{\alpha \beta}{2}, & \text{if }\,\, D_{i,j} = 2, G_{i,j} = 0, \\[1ex] \frac{\beta}{2}, & \text{if }\,\, D_{i,j} = 0, G_{i,j} = 1, \\ 1 - \beta, & \text{if }\,\, D_{i,j} = 1, G_{i,j} = 1, \\ \frac{\beta}{2}, & \text{if }\,\, D_{i,j} = 2, G_{i,j} = 1, \\ 0, & \text{if }\,\, D_{i,j} = 0, G_{i,j} = 2, \\ 0, & \text{if }\,\, D_{i,j} = 1, G_{i,j} = 2, \\ 1, & \text{if }\,\, D_{i,j} = 2, G_{i,j} = 2, \end{array}\right.  $$

where *G*_*i*,*j*_ is the unobserved true genotype at the *i*th site of cell *j*. A true homozygous non-reference genotype (site with true homozygous mutation) is affected by neither FP error nor ADO. An FN error can affect the heterozygous genotype and combined with an FP error, it can also affect the homozygous reference genotype. FP errors can affect homozygous reference genotypes.

Single-cell data sets also contain missing data, sites for which genotype information is missing. In our computation, we take Pr(*D*_*i*,*j*_|*G*_*i*,*j*_)=1 whenever *D*_*i*,*j*_=*X*. By doing so, we marginalize the effect of missing data over three possible true genotypes and this is reflected in the likelihood computation.

### Likelihood of a phylogenetic tree

#### Phylogenetic tree

We consider that the phylogenetic tree for single cells is a rooted directed binary tree $\mathcal {T} = (T, \mathbf {t})$. It has two components, a tree topology *T* and a vector of branch lengths **t**. The phylogenetic tree represents the genealogical relationship among a set of single cells. The root of this tree has homozygous reference genotypes at all sites. The leaves of the tree represent the observed single cells. The internal nodes represent ancestral cells that are not observed in the data. Cells evolve along the branches of the tree following a model of evolution and the branch length denotes the expected number of mutations per site.

#### Model of evolution

The finite-sites model of evolution for SCS data ($\mathcal {M}$) is modeled using a continuous-time Markov chain that assigns a probability to each possible transition of the genotypes. We assume that the genomic sites evolve identically and independently. Assuming three possible genotype states {0,1,2} (for ternary data) for a genomic site, the model of evolution can be represented by a 3×3 transition probability matrix. The transition probability matrix *P*_*t*_ along a branch of length *t* is computed by matrix exponentiation of the product of the transition-rate matrix (*Q*) of the Markov chain and the branch length. The entries in the transition-rate matrix denote the infinitesimal rates (during infinitesimally small time *Δ* *t*) at which the continuous-time Markov chain moves between genotype states. We also consider that the time *Δ* *t* is the smallest unit of time, during which only one event can occur at a site. Since, we are considering the somatic mutation sites, the infinitesimal rate for the genotype transition 0→1 is set to 1. This accounts for the point mutations. LOH events can result in the genotype transitions 1→0 and 1→2 whereas deletions can result in the genotype transitions 1→0, 1→2, or 2→1. To compute the infinitesimal rates for these transitions, we introduce two parameters *λ*_*d*_ and *λ*_*l*_, which account for the effects of deletions and LOH, respectively. The product of the transition-rate matrix and the branch length (*t*) is given by

5 $$ Qt = \left[\begin{array}{cccc} -t & t & 0 \\ \frac{(\lambda_{d} + \lambda_{l})\times t}{2} & -(\lambda_{d} + \lambda_{l})\times t & \frac{(\lambda_{d} + \lambda_{l})\times t}{2} \\ 0 & \lambda_{d} \times t & -\lambda_{d} \times t \end{array}\right]  $$

In Eq. 5, *Q* *t*(*i*,*j*) denotes the rate of genotype *i* changing to genotype *j* along a branch of length *t*, *i*,*j*∈{0,1,2}. *λ*_*d*_ and *λ*_*l*_ constitute the set of parameters ($\mathcal {M}_{\lambda }$) of the model of evolution.

The transition probability matrix, *P*_*t*_ is given by

6 $$ P_{t} = \exp(Qt).  $$

*P*_*t*_(*i*,*j*) denotes the probability of the transition of genotype *i* to genotype *j* along a branch of length *t*. Each entry of *P*_*t*_ is a function of *t*, *λ*_*d*_, and *λ*_*l*_.

For binary genotype states, the product of the transition-rate matrix and the branch length is given by

7 $$ Qt = \left[\begin{array}{cc} -t & t \\ \frac{(\lambda_{d} + \lambda_{l})\times t}{2} & -\frac{(\lambda_{d} + \lambda_{l})\times t}{2} \\ \end{array}\right],  $$

and the transition probability matrix is computed using Eq. 6.

#### Likelihood

Since we assume that each site evolves independently and the technical errors affect each site independently, for the observed genotype matrix given a phylogenetic tree $\mathcal {T}$, error rates ***θ***, and the parameters of the model of evolution $\mathcal {M}_{\lambda }$, the likelihood is given by

8 $$ {}\begin{aligned} \mathcal{L}(\mathcal{T}, \boldsymbol{\theta}, \mathcal{M}_{\lambda}) = \operatorname{Pr}(D|\mathcal{T}, \boldsymbol{\theta}, \mathcal{M}_{\lambda}) = \prod_{i=1}^{n} \operatorname{Pr}(D_{i}|\mathcal{T}, \boldsymbol{\theta}, \mathcal{M}_{\lambda}), \end{aligned}  $$

where *D*_*i*_ is the observed data at site *i*. This is a vector with *m* values corresponding to *m* single cells. Let *γ* be the set of possible genotypes. If *v* is an internal node of the tree with children *u*,*w*, then let $L_{i}^{v}(g), g \in \gamma $ denote the partial conditional likelihood defined by

9 $$ L_{i}^{v}(g) = \operatorname{Pr}(D_{i}^{v}| \mathcal{T}, \boldsymbol{\theta}, \mathcal{M}_{\lambda}, \hat{D_{i}}(v) = g),  $$

where $D_{i}^{v}$ is the restriction of data *D*_*i*_ to the descendants of node *v* and $\hat {D_{i}}(v)$ is the ancestral genotype for the *i*th site at node *v*. $L_{i}^{v}(g)$ is the likelihood at site *i* for the subtree rooted at node *v*, given that the genotype at *v* is *g*.

The likelihood of the complete observed data *D*_*i*_ at the *i*th site is given by

10 $$ \operatorname{Pr}(D_{i}|\mathcal{T}, \boldsymbol{\theta}, \mathcal{M}_{\lambda}) = L_{i}^{r}(0),  $$

where *r* is the root of the tree. Since we consider that the genotypes at the root are all homozygous reference (0), the probability $\operatorname {Pr}(\hat {D_{i}}(r) = 0)$ equals 1. The partial conditional likelihood function satisfies the recursive relation

11 $$ L_{i}^{v}(g) = \left[\sum_{h \in \gamma} P_{t_{vu}}(g,h)L_{i}^{u}(h)\right] \left[\sum_{h \in \gamma} P_{t_{vw}}(g,h)L_{i}^{w}(h)\right],  $$

for all internal nodes *v* with children *u* and *w*. *t*_*vu*_ and *t*_*vw*_ are the branch lengths corresponding to branches that connect *v* to *u* and *w*, respectively. $P_{t_{vu}}(g,h)$ and $P_{t_{vw}}(g,h)$ are the transition probabilities that are calculated using Eq. 6 with arguments *t*_*vu*_ and *t*_*vw*_, respectively. For a leaf of the tree that denotes a single cell *j*, the partial likelihood is given by

$$L_{i}^{j}(g) = \operatorname{Pr}(D_{i,j} | G_{i,j} = g), $$

where Pr(*D*_*i*,*j*_|*G*_*i*,*j*_) is calculated using either Eqs. 2 or 4 depending on the data. The partial likelihood values at the leaves are computed based on the error rates of the SCS data.

The log-likelihood for the observed genotype matrix given a phylogenetic tree $\mathcal {T}$, error rates ***θ***, and model parameters $\mathcal {M}_{\lambda }$ becomes a summation over *n* sites:

12 $$ \log \mathcal{L}(\mathcal{T}, \boldsymbol{\theta}, \mathcal{M}_{\lambda}) = \sum_{i=1}^{n} \log L_{i}^{r}(0).  $$

This likelihood computation uses Felsenstein’s pruning algorithm [54] for calculating the likelihood of a phylogenetic tree with the transition probabilities given by Eq. 6. To calculate the partial likelihoods for leaves, we use the SCS error model instead of values suggested in [54].

### Search algorithm to infer phylogeny

We developed a heuristic search algorithm to explore stochastically the joint space of phylogenetic trees, error rates, and evolution model parameters. In the joint $(\mathcal {T}, \boldsymbol {\theta }, \mathcal {M}_{\lambda })$ space, we need to consider three different types of moves to propose a new configuration. In tree-changing moves, a new phylogenetic tree $\mathcal {T'}$ is proposed from current state $\mathcal {T}$. In error-rate-changing moves, a new error rate ***θ*** ^***′***^ is proposed from the current error rate ***θ***. In parameter-changing modes, a new value of the parameter $\mathcal {M}_{\lambda '}$ is proposed from the current parameter value $\mathcal {M}_{\lambda }$. If the proposed configuration results in a higher likelihood, it is accepted, otherwise it is rejected.

With a small probability, the proposed configuration is accepted or rejected based on an acceptance ratio (only for tree-changing or error-rate-changing moves). The acceptance ratio for proposing a new phylogenetic tree is given by

13 $$ \rho_{T} = \min \left\{\frac{\operatorname{Pr}(D|\mathcal{T'}, \boldsymbol{\theta}, \mathcal{M}_{\lambda})q_{T}(\mathcal{T}|\mathcal{T'})}{\operatorname{Pr}(D|\mathcal{T}, \boldsymbol{\theta}, \mathcal{M}_{\lambda})q_{T}(\mathcal{T'}|\mathcal{T})}, 1 \right\},  $$

which involves calculating the ratio of the likelihood of the new configuration and the current configuration. The acceptance ratio also requires a proposal ratio, which is computed based on *q*_*T*_, the proposal distribution for proposing a new tree. A new error rate ***θ*** ^***′***^ is accepted with the ratio given by

14 $$ \rho_{\theta} = \min \left\{\frac{\operatorname{Pr}(D|\mathcal{T}, \boldsymbol{\theta'}, \mathcal{M}_{\lambda})p_{\theta}(\boldsymbol{\theta'}) q_{\theta}(\boldsymbol{\theta}|\boldsymbol{\theta'})}{\operatorname{Pr}(D|\mathcal{T}, \boldsymbol{\theta}, \mathcal{M}_{\lambda}) p_{\theta}(\boldsymbol{\theta})q_{\theta}(\boldsymbol{\theta'}|\boldsymbol{\theta})}, 1 \right\},  $$

which takes into account the ratio of the likelihoods of the new and current configurations, the ratio of the prior probability of the new and current error rates, and also a proposal ratio. *p*_*θ*_ is the prior distribution of the error rate and *q*_*θ*_ is the proposal distribution for proposing the new error rate. These steps of the search heuristic are motivated by the Metropolis–Hastings algorithm [55] for MCMC sampling and they help in exploring the likelihood space. The inference algorithm is shown in Algorithm 1.



#### Tree proposals

To explore the space of trees, we need efficient moves that can make small and big changes in the tree topology. Also, we need moves that change only the branch lengths instead of changing the topology. To ensure that our search does not get stuck in a local optimum, we use a combination of different types of moves. Lakner et al. [56] described several tree proposal mechanisms that are effective in Bayesian phylogenetic inference. Since our goal is to search the tree space effectively, we can employ the same tree proposals in our search algorithm. We adopt two different types of the tree proposals described in [56] in our search process: branch change proposals that alter branch lengths and branch-rearrangement proposals that alter the tree topology. The branch-rearrangement proposals can be divided into two subtypes: the prune and reattach moves and the swapping moves.

For proposing a new branch length, we draw a sample *u* from a uniform distribution on [0,1) and then get a random number *r* ^∗^ by applying the transformation *r* ^∗^=e^*η*(*u*−0.5)^. The new branch length *l* ^∗^ is a product of the current branch length *l* and *r* ^∗^. In this way, we update the branch length of all branches. This ensures that the branch lengths are locally changed and the proposal ratio becomes a product $\prod _{k}r^{*}_{k}$, where *k* is the total number of branches in the tree. *η* is a tuning parameter that is set to the value suggested in [56].

We consider two types of pruning-regrafting moves, namely random subtree pruning and regrafting (rSPR) and extending subtree pruning and regrafting (eSPR), which were described in [56]. The pruning-regrafting moves randomly select an interior branch, prune a subtree attached to that branch, and then reattach the subtree to another regrafting branch present in the other subtree. For rSPR, the regrafting branch is chosen randomly. For eSPR, an extension probability guides the movement of the point of regrafting across one branch at a time. eSPR favors local rearrangements more.

We consider three types of swapping moves, namely stochastic nearest-neighbor interchange (stNNI), random subtree swapping (rSTS), and extending subtree swapping (eSTS). stNNI chooses an internal branch as the focal branch and stochastically swaps the subtrees attached to the focal branch. eSTS also involves the swapping of two subtrees but not necessarily nearest neighbors. The subtrees are chosen according to an extension mechanism like eSPR. For rSTS, two randomly chosen subtrees are swapped.

At each step of the search algorithm, one of these six moves is chosen with a fixed probability. The proposal ratio associated with each branch-rearrangement proposal is described in detail in [56].

#### Estimation of error rate

During the search process, we also update error rates. The estimates of error rates that are input to SiFit are used to design the prior probability *p*(*θ*). The error rate being a probability (value between 0 and 1), we choose a beta prior. The mean of the prior is estimated from the input error rate and observed genotype matrix. We choose a large standard deviation to cover a wide range of values. We choose a normal distribution as the proposal distribution for proposing the new error rate. At each generation, the normal distribution is centered on the current value of the error rate. A user-specified fixed probability determines whether, in a particular iteration, a new error rate will be proposed.

#### Estimation of parameters of model of evolution

The parameters of the model of evolution, *λ*_*d*_ and *λ*_*l*_, are also updated during the search process. For each of these parameters, the next value is proposed from a normal distribution centered at the current value. The standard deviation is chosen so that a wide range of values are covered. These parameters being relative quantities (they denote the rates of deletion and LOH, respectively, relative to the rate of point mutations), we choose a beta distribution as their prior. Like proposing new error rates, a user-specified fixed probability determines whether, in a particular iteration, a new value of these parameters will be proposed.

#### Complexity analysis

In each step of the algorithm, finding the likelihood of the tree is the most expensive task. For *m* single cells and *n* sites, the likelihood calculation takes $\mathcal {O}(mk^{2}n)$, where *k* is the maximum number of states per site. For genotype data, *k*=3 and for a binary mutation matrix, *k*=2.

The number of iterations used in SiFit is user-defined. Assuming *i* to be the number of iterations used for running SiFit, the overall complexity becomes $\mathcal {O}(mk^{2}ni)$.

### Tree inference error metric

To measure the accuracy of tree inference, we used a metric that compares the topology of the inferred tree to that of the true tree and computes a distance between the two. This metric was proposed for general phylogenetic trees in [46] and it is based on the symmetric difference between the bipartitions of the two trees. The topology of a tree can be represented by the bipartitions present in the tree. A bipartition of a tree based on an edge gives us two set of leaves that would be formed by deleting the edge. If $\mathcal {E}$ is the set of edges of $\mathcal {T}$, then the bipartition encoding of $\mathcal {T}$, denoted by $C(\mathcal {T}) = \{\xi (e): e \in \mathcal {E}\}$, is the set of bipartitions defined by each edge in $\mathcal {T}$. *ξ*(*e*) is the bipartition on the leaf set of $\mathcal {T}$ produced by removing the edge *e* from $\mathcal {T}$. We consider three distances between two trees.

If $\mathcal {T}_{t}$ is the true tree on a set of single cells $\mathcal {S}$ and $\mathcal {T}_{i}$ is the inferred tree, then the following are the three inference error metrics:

1. *False negative (FN) distance*: This counts the edges in $\mathcal {T}_{t}$ that induce bipartitions that are not present in $C(\mathcal {T}_{i})$. This distance is normalized by dividing by the total number of bipartitions in $\mathcal {T}_{t}$, i.e., ${\left |{C(\mathcal {T}_{t})\setminus C(\mathcal {T}_{i})}\right |}/{\left |{C(\mathcal {T}_{t})}\right |}$.
2. *False positive (FP) distance*: This counts the edges in $\mathcal {T}_{i}$ that induce bipartitions that are not present in $C(\mathcal {T}_{t})$. This distance is normalized by dividing by the total number of bipartitions in $\mathcal {T}_{i}$, i.e., ${\left |{C(\mathcal {T}_{i})\setminus C(\mathcal {T}_{t})}\right |}/{\left |{C(\mathcal {T}_{i})}\right |}$.
3. *Robinson–Foulds (RF) distance*: The Robinson–Foulds distance is the average of the FP and FN distances. This is the most common error metric.

If the two trees to compare are binary, then we use the RF distance between them as the error metric. For binary trees, the FP, FN, and RF distances are equal to each other. To compare a true binary tree to an inferred non-binary tree, we compute the FP and FN distances separately.

SiFit, SCITE, and MrBayes output a binary tree that can be compared against the true tree in terms of RF distance. For OncoNEM, we consider the cell lineage tree that it infers, which we convert into an equivalent phylogenetic tree by projecting the observed single cells to leaves (shown in Additional file 1: Figure S11). The equivalent phylogenetic tree might be binary or non-binary and we compute both the FP and FN distances for it when comparing to the true tree.

### Inference of ancestral sequences and order of mutations

Inferring the chronological order of mutations in the tumor lineage requires inferring the mutation status of the internal nodes so that the mutations can be placed on the branches of the phylogeny. We infer the mutational profiles of the internal nodes using a likelihood-based approach that finds the most likely mutational profile for an internal node given the phylogenetic tree and error rates. We extend the dynamic programming algorithm for inferring ancestral sequences described in Pupko et al. [48] to account for the error rates of the single cells.

For a single cell *c* at the leaf of the tree, the partial likelihood for a genotype *g* at site *i* is calculated as

$$L_{c}(g) = \underset{h} {\operatorname{argmax}} \, P_{t_{vc}}(g,h) \operatorname{Pr} (D_{i,c}|G_{i,c} = h). $$

The mutation state *m*_*c*_(*g*) is set to the value of *h* that attains the maximum value for partial likelihood. *v* is the parent of *c* and *t*_*vc*_ is the branch length connecting *v* to *c*. For a missing data point, Pr(*D*_*i*,*c*_|*G*_*i*,*c*_=*h*) becomes 1. For a non-root internal node, *u*, with children *y* and *z*, the partial likelihood is calculated as

$$L_{u}(g) = \underset{h} {\operatorname{argmax}} \, P_{t_{wu}}(g,h)L_{y}(h)L_{z}(h). $$

The mutation state *m*_*u*_(*g*) is set to the value of *h* that attains the maximum value. For the root of the tree, mutation state *m*_*r*_=0 and the mutation state for an internal node, *u*, whose parent *w*’s mutation state is already determined as *g*, is chosen as *m*_*u*_(*g*).

After inferring the mutational profiles of the internal nodes, the mutations on a branch can be found by finding the SNV sites for which the mutational status of the two nodes at the two ends of the branch differs.

### Clustering of cells

To cluster the cells into subpopulations for the tumor data sets, we used *k*-medoids clustering with silhouette scores. A distance matrix was obtained for the cells containing mutations from the ML tree reconstructed by SiFit, in which an entry represents the distance between two cells. The distance between two cells was calculated by summing the branch lengths on the path that connects the two cells. *k*-medoids clustering was performed on the resulting distance matrix using the clustering library of R (http://www.r-project.org) and the number of clusters was varied from 2 to 5. In each case, the average silhouette score was measured and the number of clusters that maximized the silhouette score was reported as the optimal number of clusters.

### Simulation of synthetic data

#### Evolution of single-cell sequences

To simulate single-cell data sets, first, a random binary tree is constructed on a leaf set of single cells by a recursive algorithm that randomly divides the set of cells into two subtrees that are also randomly generated, and then joins them into a single tree by choosing a root that has the two subtrees as the left and right children. We specify the number of sites *n* in the single-cell genome. The root node of the phylogeny is populated with a homozygous reference genotype (*g*=0) at each site. In each branch of the tree, a Poisson-distributed number of sites *p* is mutated. If *t* is the branch length, the parameter for the Poisson distribution is chosen as *t*×*n*, so that on average, a child node in the tree differs from its parent by the proportion of loci, which is given by the branch length. When mutating a new site, the genotype changes from a homozygous reference (*g*=0) to heterozygous (*g*=1). Recurrent mutations are introduced with probability *r*. If the locus in the node for which a recurrent mutation happens has a homozygous reference genotype (*g*=0), then a parallel mutation happens in that branch, i.e., the genotype changes from a homozygous reference (*g*=0) to heterozygous (*g*=1). If the locus in the node already contains a mutated genotype, then a back-mutation results in reverting the genotype to the homozygous reference (*g*=0). To simulate LOH events, the loci with heterozygous (*g*=1) genotypes are set to either homozygous reference (*g*=0) or homozygous non-reference (*g*=2) genotypes with probability *ω*. If LOH happens at a locus, either of the homozygous genotypes are chosen with equal probability. A deletion is simulated with probability *d* at a branch. A deletion can affect multiple loci at a time. For a heterozygous site, a deletion can happen for any of the copies, resulting in either of the homozygous genotypes (*g*=0 or *g*=2). A deletion does not affect the homozygous reference genotypes but can change the homozygous non-reference genotypes to a heterozygous genotype. In this way, sites are evolved at each branch of the tree. At the corner case, when there is no new locus to mutate at a branch, recurrent mutations are introduced. After considering all the branches of the tree, we have the single-cell genotypes at the leaves of the tree.



#### Simulating doublets

Doublets are events when two cells get trapped in the same well, resulting in a merger of the genotypes of the two cells. To model doublets, we need to define the expected genotype state, which is a combination of two genotype states. The expected genotype state can be defined by a binary operator ⊕ whose results for SNV data are shown in Table 1. *δ* denotes the fraction of cells that are doublets. With probability *δ*, a cell is chosen to be a doublet and its genotype is combined with that of a randomly sampled co-trapped cell (the genotype of which is a copy of that of another cell in the tree) to form the new genotype as defined by the ⊕ operator. The pseudocode for simulating doublets is shown in Algorithm 2.

**Table 1:** Expected genotype state after combining two genotypes using the binary operator ⊕

⊕	*g*=0	*g*=1	*g*=2
*g*=0	0	1	1
*g*=1	1	1	1
*g*=2	1	1	2

## Additional file

Additional file 1 Supplementary Material. This file contains a supplementary note and supplementary figures. (PDF 1024 kb)
