# Supplementary material for: SiFit: inferring tumor trees from single-cell sequencing data under finite-sites models
Source: Genome Biol. 2017 Sep 19;18:178. doi: 10.1186/s13059-017-1311-2 (PMC5606061; doi:10.1186/s13059-017-1311-2)
Supplement: Supplementary file 1 — Supplementary Material. This file contains a supplementary note and supplementary figures. (PDF 1024 kb) [file 13059_2017_1311_MOESM1_ESM.pdf]

Supplementary material for "SiFit: A Method for Inferring Tumor Trees from Single-Cell Sequencing Data under Finite-site Models"

Hamim Zafar[1,2] , Anthony Tzen[1] , Nicholas Navin[2,3] , Ken Chen[*2] and Luay Nakhleh[*1]

[1]Department of Computer Science, Rice University, Houston, Texas, USA [2]Department of Bioinformatics and Computational Biology, the University of Texas M.D. Anderson Cancer Center, Houston, Texas, USA. [3]Department of Genetics, the University of Texas M.D. Anderson Cancer Center, Houston, Texas, USA.

Email: Hamim Zafar - hz22@rice.edu; Anthony Tzen - ayt1@rice.edu; Nicholas Navin - NNavin@mdanderson.org; Ken Chen*- KChen3@mdanderson.org; Luay K Nakhleh*- nakhleh@rice.edu;

*Corresponding author

Supplementary Note

Potential events violating Four-gamete test in single-cell sequencing data

The four-gametes theorem states that an $m \times n$ binary matrix, M, has an undirected perfect phylogeny if and only if no pair of columns contain all four binary pairs $(0,0;\ 0,1;\ 1,0$ and $1,1)$, where m represents the number of taxa (leaves of the tree) and n represents genomic sites. A perfect phylogeny represents a rooted tree (T) on a leafset of m taxa and each of the n genomic sites (characters) labels exactly one edge of T. The genomic sites that are mutated in a particular taxa (c) are the genomic sites that label the branches along the unique path from the root to the leaf (labeled by c) of the perfect phylogeny tree T. The perfect phylogeny model conveys that each genomic site represents a perfect character, i.e. each site mutates exactly once in the evolutionary history of the character. This assumption in other words is known as "infinite sites assumption". A binary matrix that maintains the four-gamete condition can be thought of as following infinite-sites model of evolution and any violation of the four-gamete condition can suggest potential deviation from "infinite sites assumption".

In single-cell sequencing binary genotype data, several events can lead to violation of four-gamete test.

- **Mutations affecting the same site** Different mutation events in cancer such as deletion, LOH and convergent evolution can mutate a genomic site more than once. This will make that particular site 'imperfect' resulting in a violation of four-gamete condition (see Fig. S5b for an example).

- **Cell doublets** 'Cell doublets' are formed when two or more cells are accidentally isolated instead of single cells. This results in merging the genotype of two or more cells. The merging of genotype can also lead to violation of four-gamete condition (see Fig. S5c for an example).

- **False positive and false negative errors** In SCS data, false positive and false negative errors can lead to violation of four-gamete condition (see Fig. S5d for an example) as the false positive errors are random and the false negative errors create the same effect for heterozygous sites as back mutations.

All the above mentioned factors can occur together resulting in a huge number of pairs of genomic sites violating four-gamete test. Fig. S6 shows the relative contribution of each of these factors in violating four-gamete test. Results are averaged over 10 datasets. The datasets were generated with the following values of the parameters, $m = 100$, $n = 200$, $\delta = 0.1$, $\alpha = 0.01$ and $\beta = 0.2$. All factors together could result in the final number of pairs of sites in violation of the four-gamete condition.

Supplementary Figures

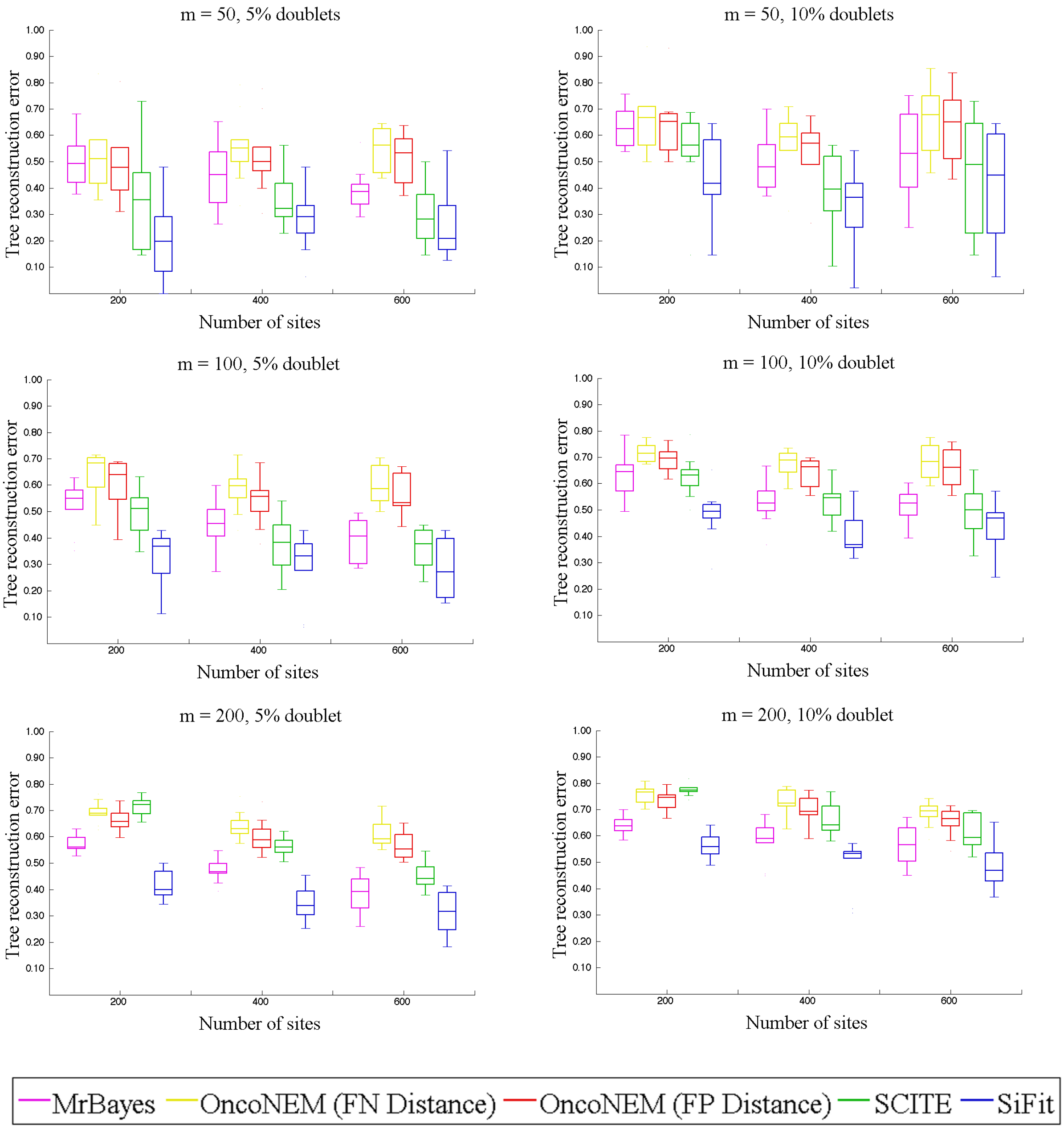

Figure S1: Performance comparison on datasets (containing doublets) with varying number of cells. SiFit's tree reconstruction accuracy is compared against that of SCITE, OncoNEM and MrBayes. The y-axis denotes the tree reconstruction error that measures the distance of the inferred tree from the ground truth. All cells including the doublets are considered for measuring the tree reconstruction error. The number of cells varies as $m = 50$, $m = 100$ and $m = 200$. The percentage of doublets (δ) varies as 5% and 10%. For each combination of δ and m, the number of sites n is varied as $m = 200$, $m = 400$ and $m = 600$. Each boxplot summarizes result over 10 datasets for each combination of δ, n and m.

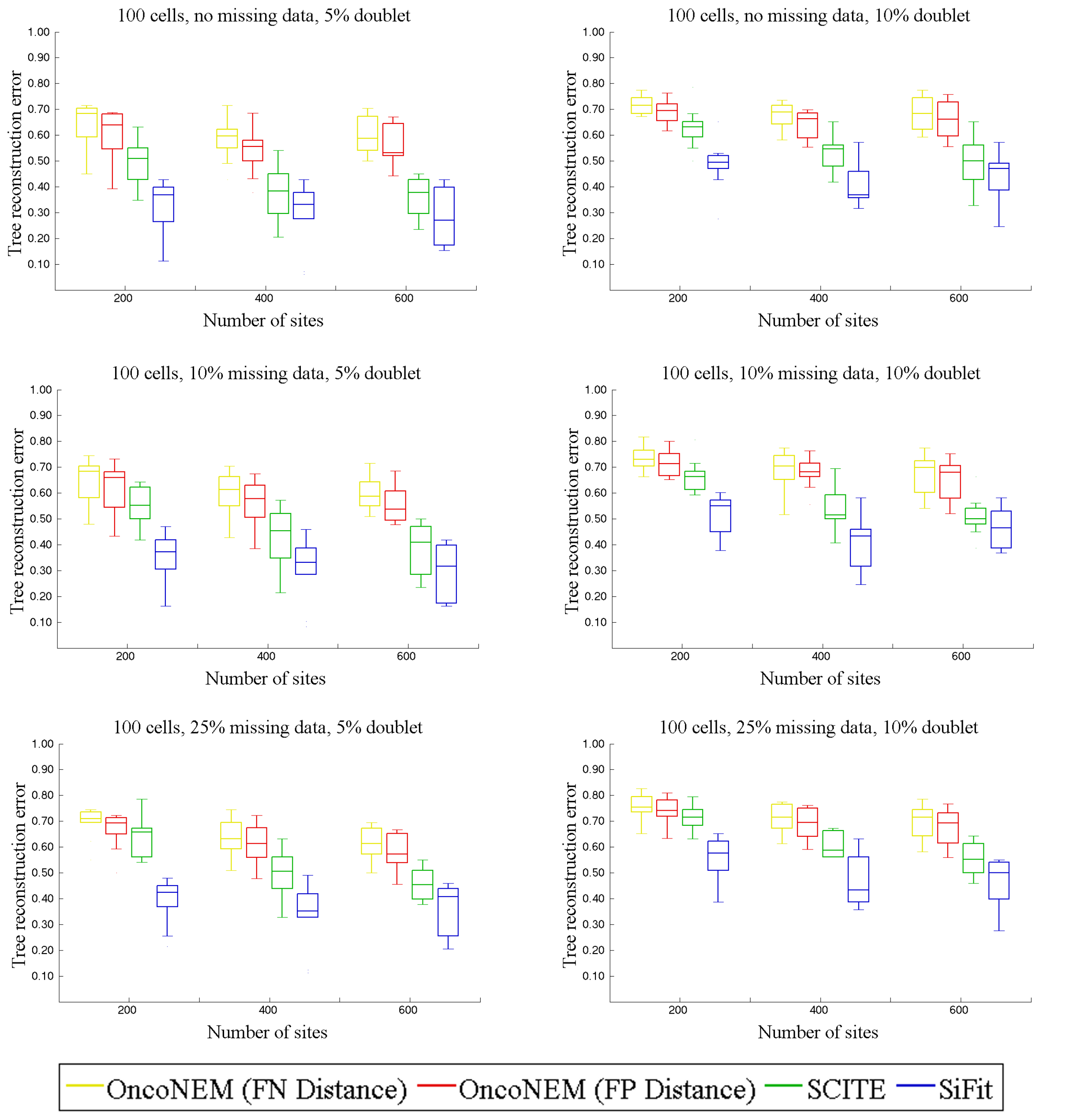

Figure S2: Performance comparison on datasets (containing doublets) with missing data. SiFit's tree reconstruction accuracy is compared against that of SCITE and OncoNEM . The y-axis denotes the tree reconstruction error that measures the distance of the inferred tree from the ground truth. All cells including the doublets are considered for measuring the tree reconstruction error. The amount of missing data varies from $\{0\%, 10\%, 25\%\}$. The percentage of doublets (δ) varies as 5% and 10%. For each combination of δ and percentage of missing data, the number of sites n is varied as $m = 200$, $m = 400$ and $m = 600$. Each boxplot summarizes result over 10 datasets for each combination of δ, n and missing data percentage.

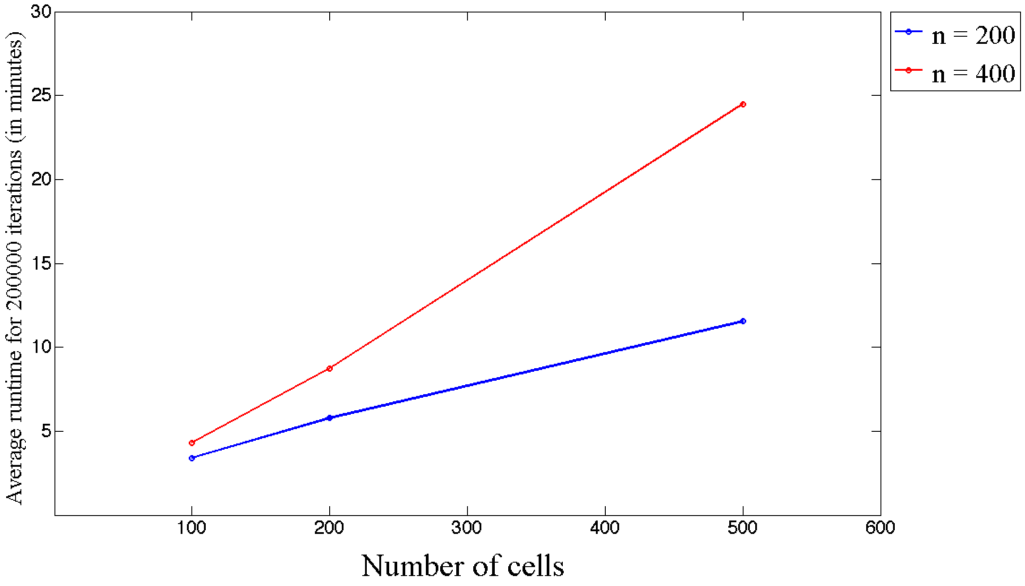

Figure S3: Scalability of SiFit. The average time for running 200,000 iterations of SiFit is recorded as the number of cells in the tree is varied along the x-axis. The number of sites in the genotype matrix is also varied.

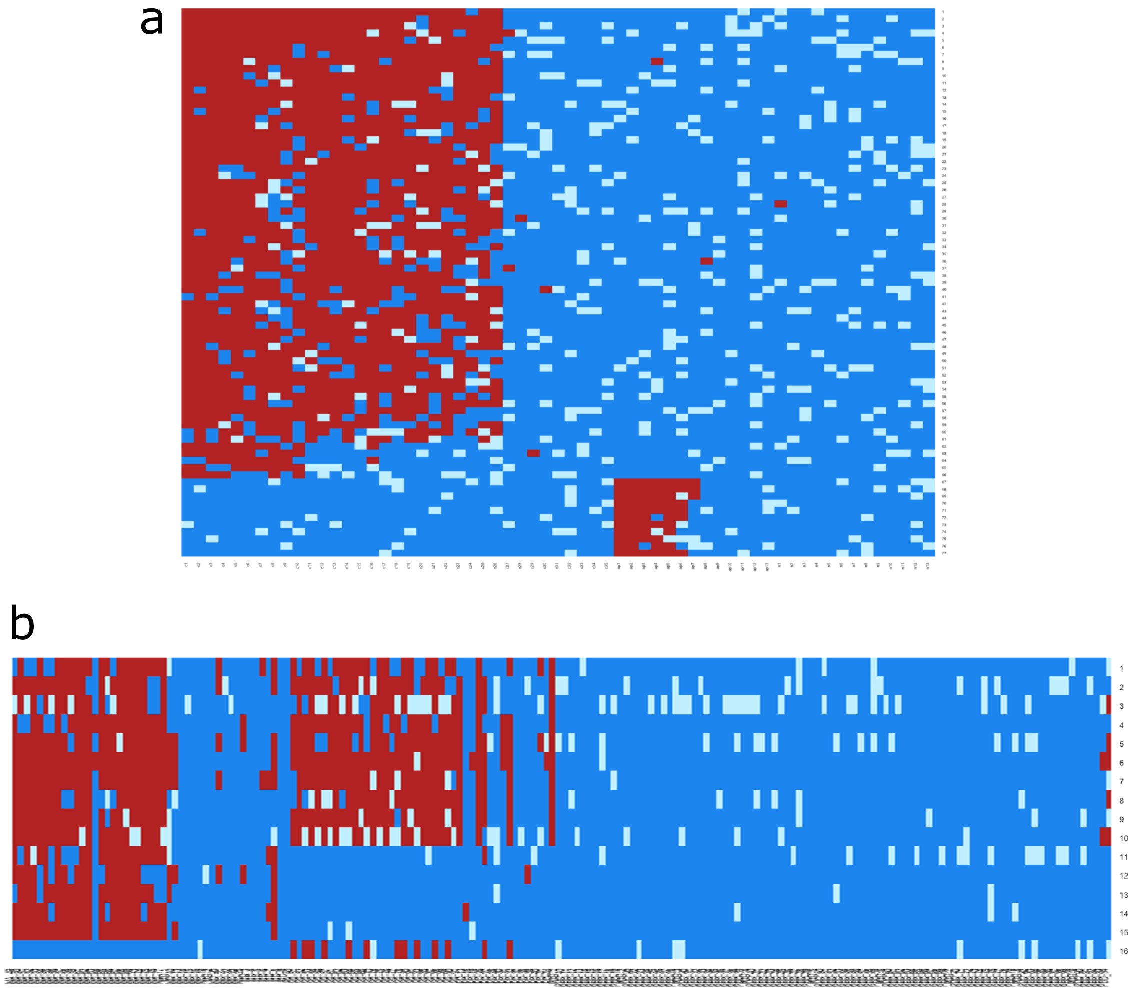

Figure S4: Mutation matrices for experimental datasets used in this study. (a) Observed genotype matrix from exome-sequencing data from adenomatous polyps and cancer tissue of a non-hereditary colorectal cancer patient. The matrix consists of 77 somatic single-nucleotide variants (SNVs) from 61 cells. (b) Observed genotype matrix from high-throughput single-cell sequencing data from a metastatic colorectal cancer patient. The matrix consists of 16 somatic single-nucleotide variants (SNVs) from 178 cells. Color coding of matrix cells: red - mutation present in the cell, deep blue - mutation not present in the cell (homozygous reference genotype), light blue - missing data.

a

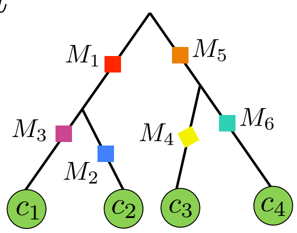

	M_1	M_2	M_3	M_4	M_5	M_6
c_1	1	0	1	0	0	0
c_2	1	1	0	0	0	0
c_3	0	0	0	1	1	0
c_4	0	0	0	0	1	1

b

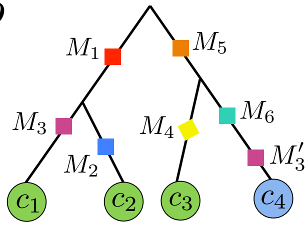

	M_1	M_2	M_3	M_4	M_5	M_6
c_1	1	0	1	0	0	0
c_2	1	1	0	0	0	0
c_3	0	0	0	1	1	0
c_4	0	0	1	0	1	1

c

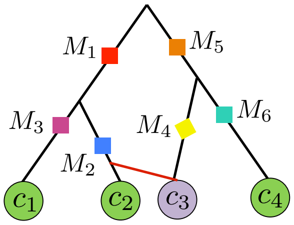

	M_1	M_2	M_3	M_4	M_5	M_6
c_1	1	0	1	0	0	0
c_2	1	1	0	0	0	0
c_3	1	1	0	1	1	0
c_4	0	0	0	0	1	1

d

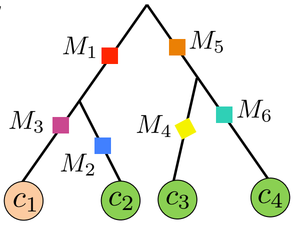

	M_1	M_2	M_3	M_4	M_5	M_6
c_1	1	0	1	0	0	1
c_2	1	1	0	0	0	0
c_3	0	0	0	1	1	0
c_4	0	0	0	0	1	1

Figure S5: Illustration of potential events violating four-gamete test. a) A perfect phylogeny with 4 cells as leaves. The green circles are the cells, the colored diamonds are the mutations. Corresponding binary mutation matrix is shown in the right. b) Violation of four-gamete principle due to mutations (deletion, LOH and recurrent point mutations) affecting the same site. The mutation M_3 occurs again in the cell c_4 (marked in blue). Columns M_1 and M_3 (highlighted in red) violate four-gamete principle. c) Violation due to cell doublets. The cell c_3 (marked in purple) is a doublet now and its genotype is merged now with a cell having same genotype as c_2. The columns M_2 and M_5 (highlighted in red) violate four-gamete principle. d) Violation due to amplification error. The cell c_1 (marked in orange) has a FP error for mutation M_6. The columns M_1 and M_6 (highlighted in red) violate four-gamete principle.

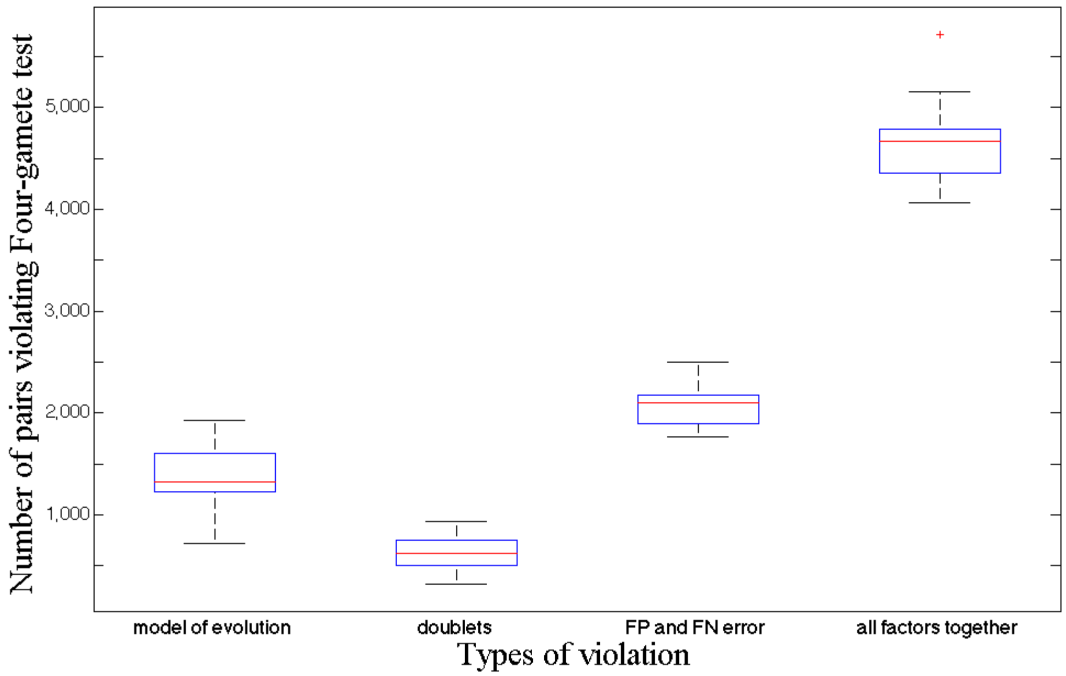

Figure S6: Potential events violating four-gamete test. Number of pairs of genomic sites violating four-gamete test due to events such as recurrent mutations, cell doublets and FP and FN error. Results for $m = 100$, $n = 200$, $\delta = 0.1$, $\alpha = 0.01$ and $\beta = 0.2$ are averaged over 10 datasets, y-axis represents the number of pairs violating four-gamete test

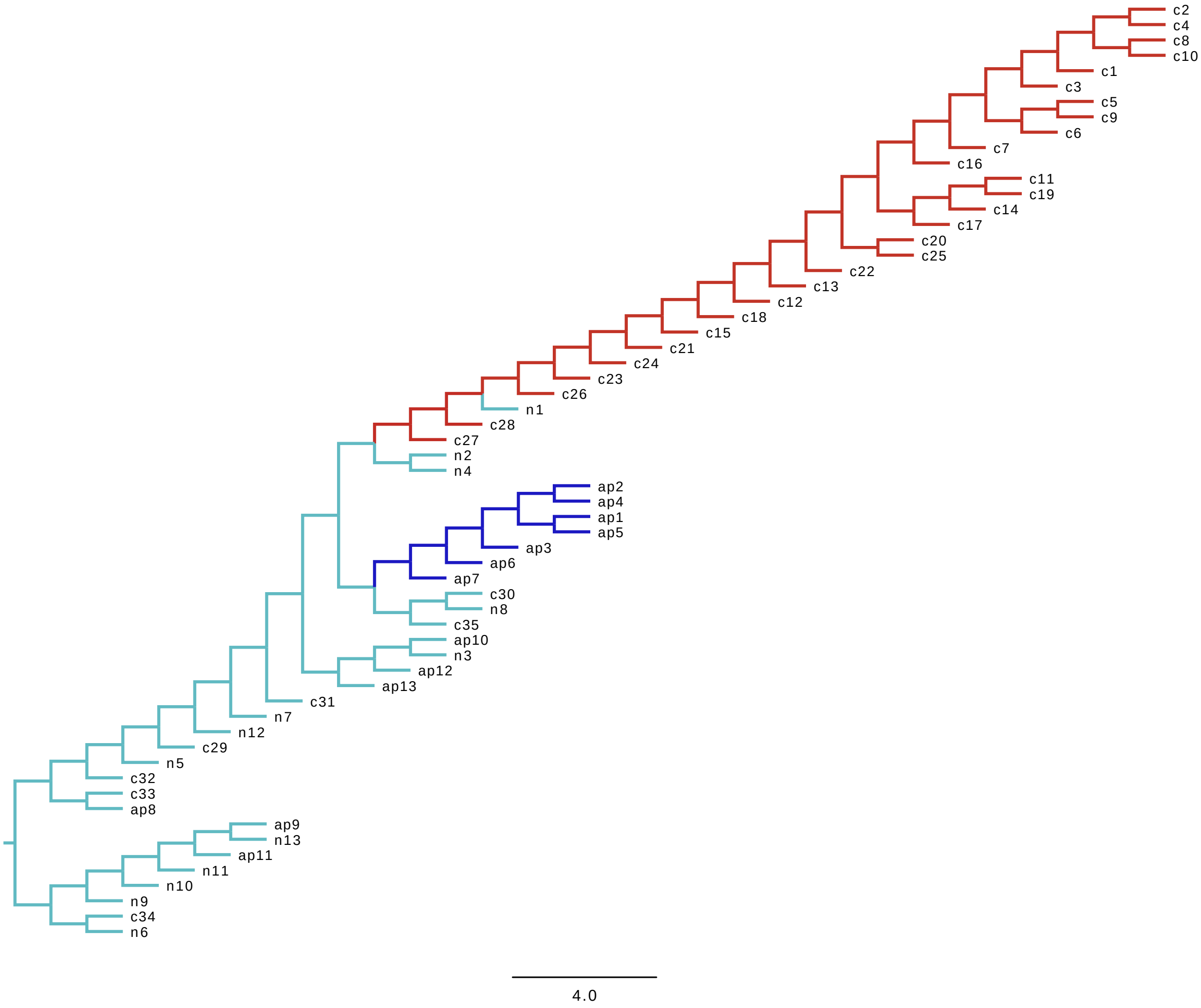

Figure S7: Maximum Likelihood tree reconstructed by SCITE for non-hereditary colorectal cancer patient. The cyan branches have cells without mutation as leaves. The red branches are connected to single tumor cells as leaves. The blue branches are connected to adenomatous polyp cells as leaves.

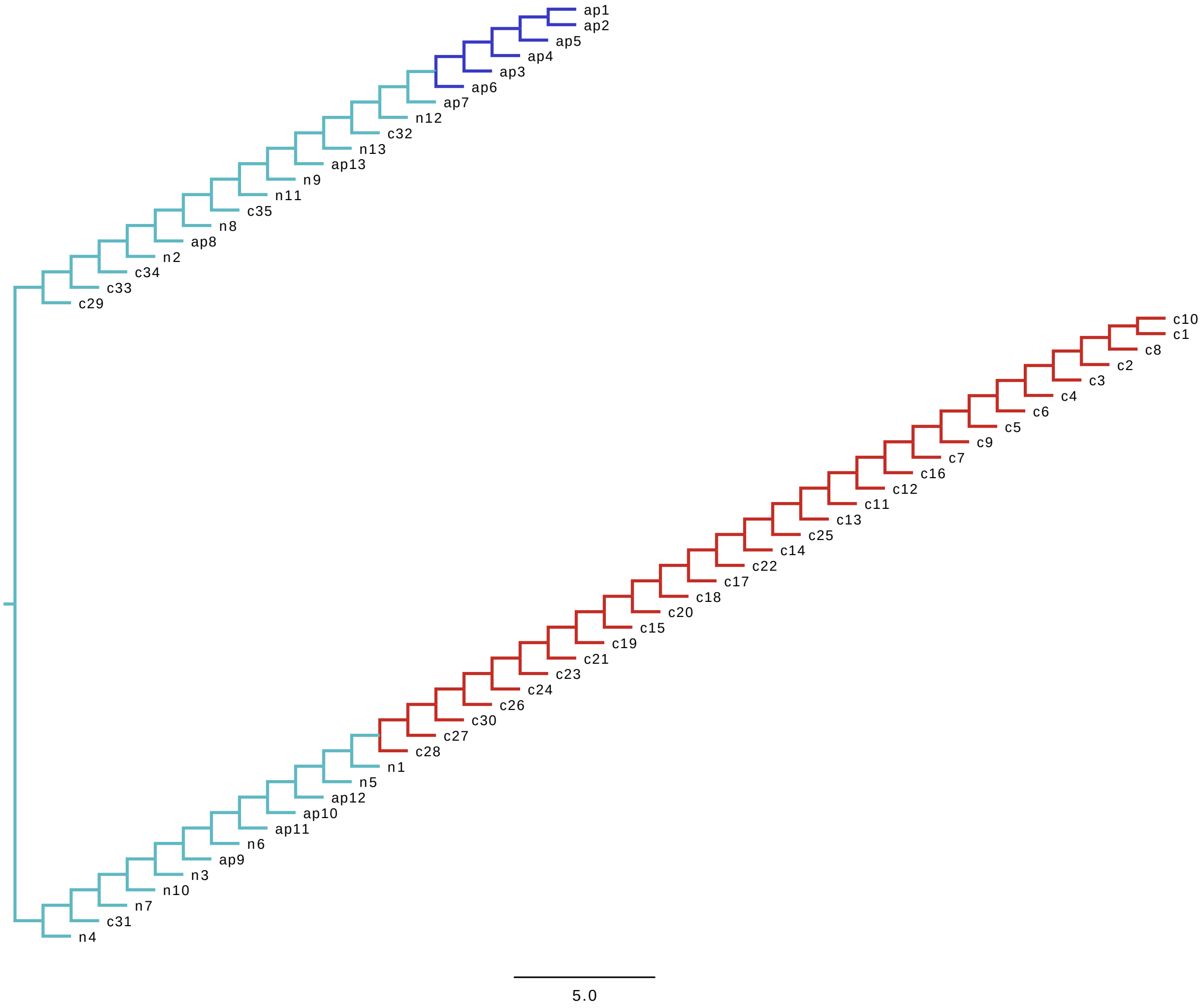

Figure S8: Maximum Likelihood tree reconstructed by OncoNEM for non-hereditary colorectal cancer patient. The cyan branches have cells without mutation as leaves. The red branches are connected to single tumor cells as leaves. The blue branches are connected to adenomatous polyp cells as leaves.

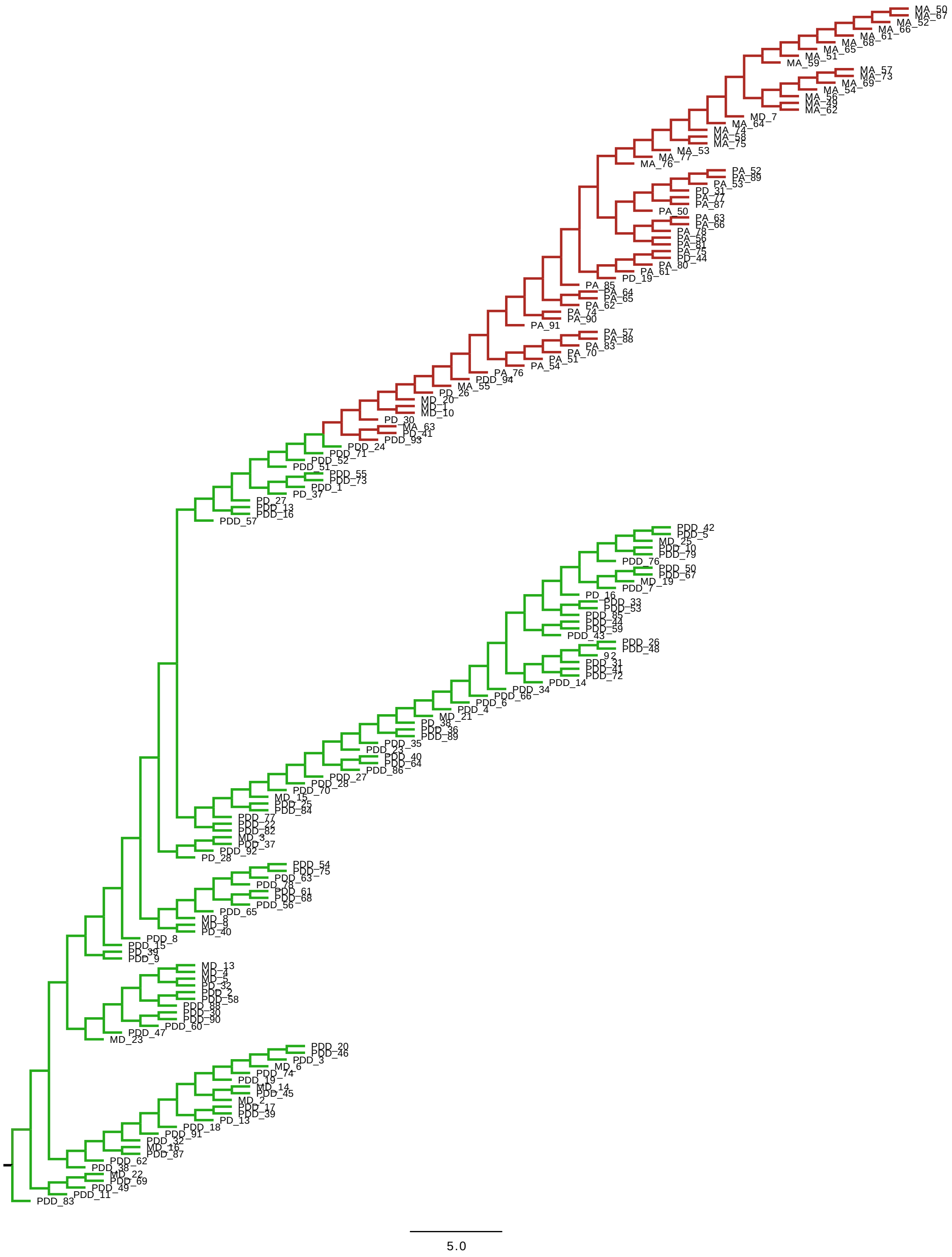

Figure S9: Maximum Likelihood tree reconstructed by SCITE for metastatic colorectal cancer patient. The green branches have normal cells without mutation as leaves. The red branches are connected to single tumor cells as leaves.

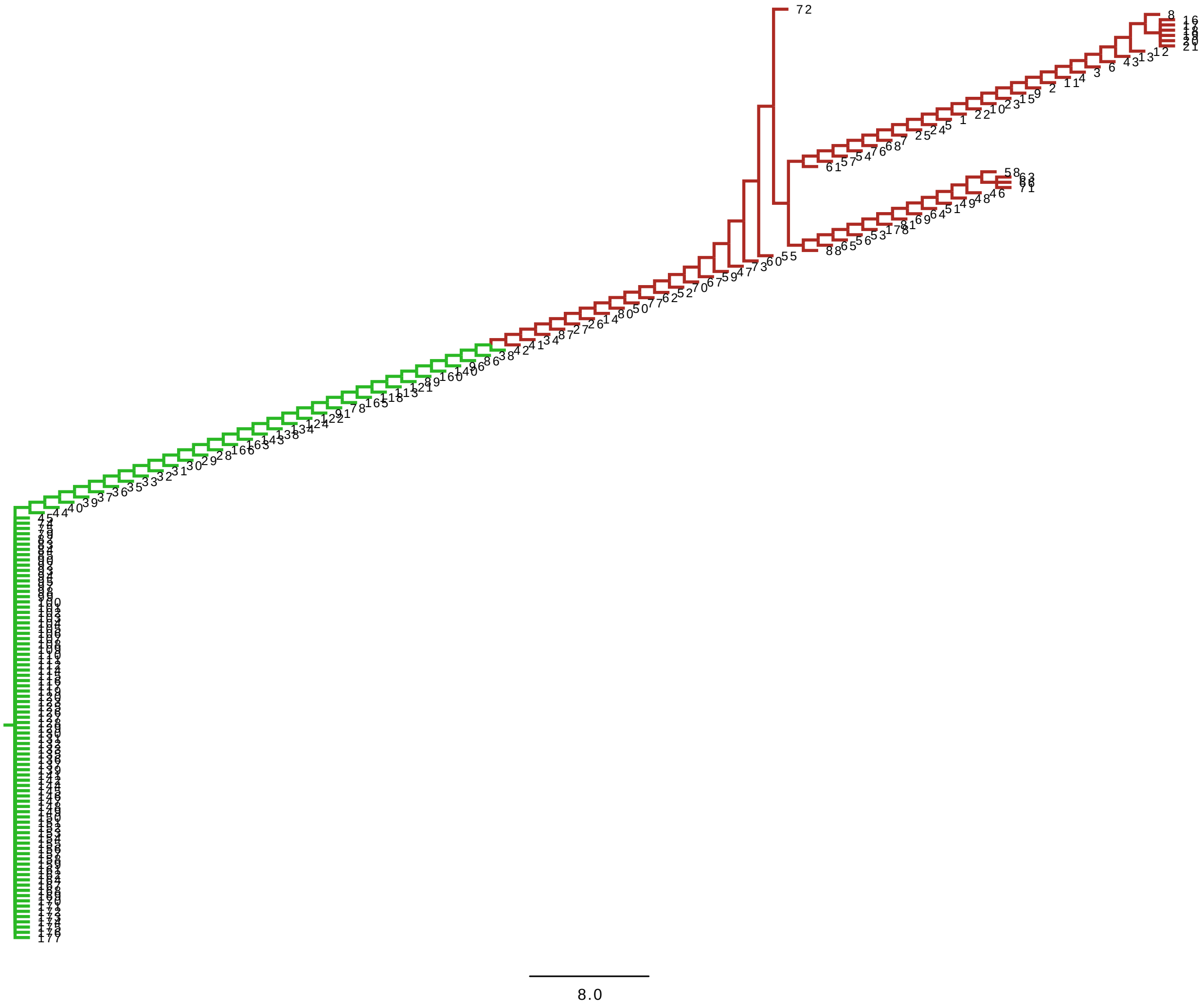

Figure S10: Maximum Likelihood tree reconstructed by OncoNEM for metastatic colorectal cancer patient. The green branches have normal cells without mutation as leaves. The red branches are connected to single tumor cells as leaves.

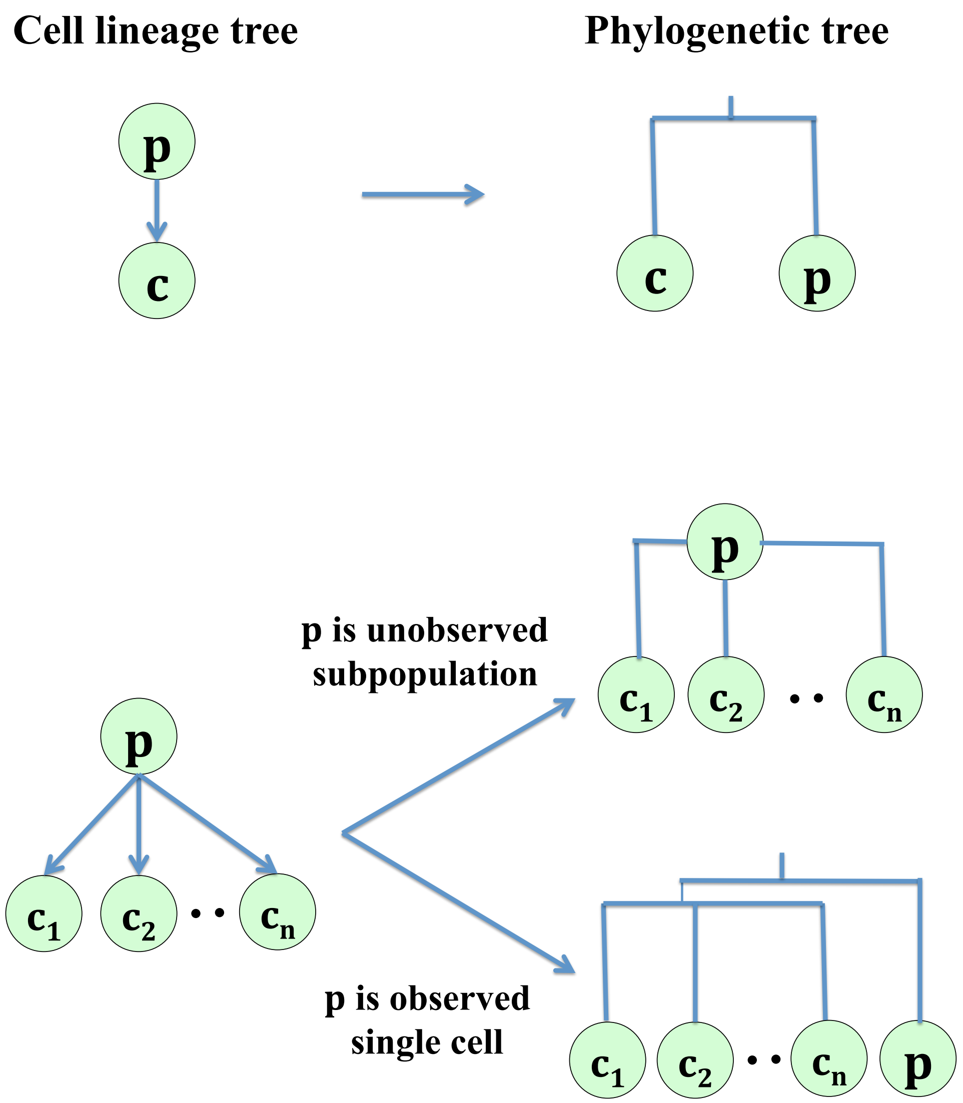

Figure S11: Conversion of cell lineage tree inferred by OncoNEM to equivalent phylogenetic tree. Cell lineage tree has two basic types of components. The equivalent phylogenetic tree component is shown on the right.
